# Prospects for More Efficient Multi-Photon Absorption Photosensitizers Exhibiting Both Reactive Oxygen Species Generation and Luminescence

**DOI:** 10.3390/molecules26206323

**Published:** 2021-10-19

**Authors:** Emma Robbins, Stéphanie Leroy-Lhez, Nicolas Villandier, Marek Samoć, Katarzyna Matczyszyn

**Affiliations:** 1 Laboratoire PEIRENE, Université de Limoges, 123 Avenue Albert Thomas, 87060 Limoges, France; emma.robbins@etu.unilim.fr (E.R.); stephanie.lhez@unilim.fr (S.L.-L.); nicolas.villandier@unilim.fr (N.V.); 2 Advanced Materials Engineering and Modelling Group, Faculty of Chemistry, Wrocław University of Science and Technology, Wybrzeże Wyspianskiego 27, 50-370 Wrocław, Poland; marek.samoc@pwr.edu.pl

**Keywords:** photosensitizers, multi-photon absorption, two-photon absorption, reactive oxygen species, photodynamic therapy, nanoparticles, gold nanoparticles

## Abstract

The use of two-photon absorption (TPA) for such applications as microscopy, imaging, and photodynamic therapy (PDT) offers several advantages over the usual one-photon excitation. This creates a need for photosensitizers that exhibit both strong two-photon absorption and the highly efficient generation of reactive oxygen species (ROS), as well as, ideally, bright luminescence. This review focuses on different strategies utilized to improve the TPA properties of various multi-photon absorbing species that have the required photophysical properties. Along with well-known families of photosensitizers, including porphyrins, we also describe other promising organic and organometallic structures and more complex systems involving organic and inorganic nanoparticles. We concentrate on the published studies that provide two-photon absorption cross-section values and the singlet oxygen (or other ROS) and luminescence quantum yields, which are crucial for potential use within PDT and diagnostics. We hope that this review will aid in the design and modification of novel TPA photosensitizers, which can help in exploiting the features of nonlinear absorption processes.

## 1. Introduction

### 1.1. TPA and Its Applications

**Figure 1:**
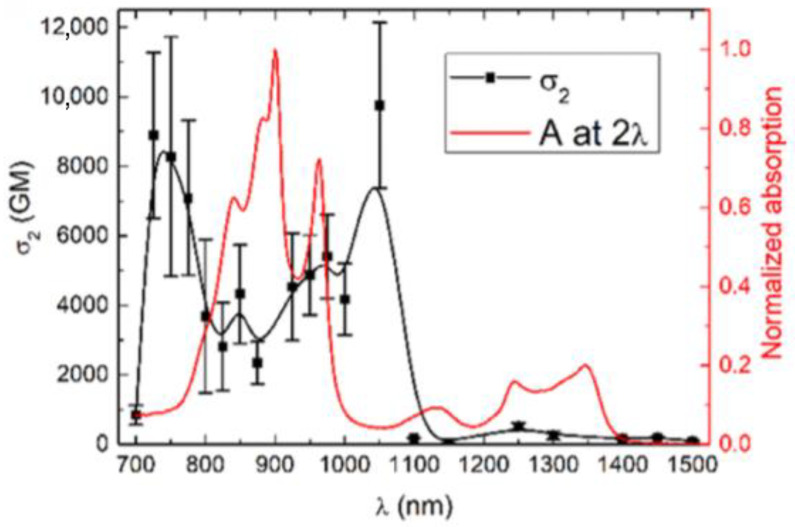
An example of a comparison of a TPA spectrum with one-photon absorption. The red curve represents the linear, one-photon absorption plotted at twice the wavelength, overlaid with the two-photon absorption cross-section spectrum (**16**, Section 4.1) [22]. Figure used with permission from © Mazur, et al.

TPA is the simultaneous absorption of two photons by the same species (e.g., molecule, nanoparticle, crystal). The combined energy of the two photons equals the transition energy [1]. The final state that is reached may be the same as that accessible through one-photon transition, but this is not always the case. Figure 1 displays an example of a two-photon absorption spectrum, i.e., the dependence of the TPA cross-section on the excitation wavelengths overlaid with one-photon absorption plotted against twice the excitation wavelength. Notably, the two curves do not overlap, which is typical for centrosymmetric molecules. It should be noted that two-photon absorption can also occur in a step-wise manner (with participation of an intermediate excited state), which is not discussed herein in detail; that process has been the topic of a recent review [2]. TPA was first theoretically analyzed by Maria Göppert-Mayer (the two-photon absorption cross-section unit is named after her, 1 GM = 10^−50^ cm^4^s) in the 1930s [3]; however, it was not until the development of the laser that two-photon absorption was experimentally observed in CaF_2_:Eu^2+^ crystal in 1961 [4]. With the developments and increased availability of short pulse (picosecond and femtosecond) lasers, the studies of TPA became more widespread and detailed in the 1990s, and much information has been collected on the materials that provide effective TPA. Many applications of this effect, such as those in photodynamic therapy (PDT) [5,6,7], optical data storage [8,9], optical power limiting [10], imaging [11,12], 3D photopolymerization [13,14], microfabrication and lithography [15,16,17], photodiodes [18,19], and laser pulse characterization [20] have been suggested. Applications of TPA exploit several features of this phenomenon. First of all, TPA uses photons of lower energy than the usual one-photon absorption process; often, it is possible to reach the same excited state by two photons at twice longer wavelength (e.g., 800 nm) than that for one-photon excitation (e.g., 400 nm). This may provide better penetration of the exciting beam due to less absorption and scattering. The fact that the transition probability for the TPA process is dependent on the square of the intensity of the light beam used for the excitation (in the most common case of degenerate TPA, i.e., the two absorbed photons having the same energy), allows one to spatially control the excitation of a specimen in three-dimensions, to the region tightly confined to the vicinity of the focus of the beam [1,21].

**Figure 2:**
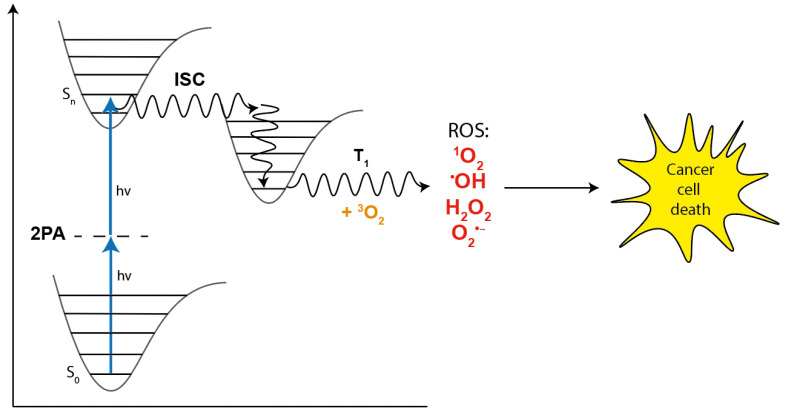
Scheme of TPA driven PDT. The depicted S_0_ → S_n_ transition must be two-photon allowed, which is generally the case for non-centrosymmetric species (in such a case it is possible for *n* = 1). In the presence of a center of symmetry, the one- and two-photon transitions are mutually exclusive, thus not all excited states are available for the TPA process (and often *n* = 2) [29].

We focus in this review on the special kind of two-photon absorbing species that are called photosensitizers. These are basically molecules, or more complicated systems such as nanoparticles, that are capable of producing so-called reactive oxygen species (ROS) that may be used in biological applications including Photodynamic Therapy (PDT). PDT is a non-invasive procedure used in the treatment of certain cancers that utilizes three main components: a light source, a photosensitizer (PS), and molecular oxygen that is present in the tissue. The light source is used to activate the PS (usually an organic or organometallic molecule) to its excited state, which can then interact with molecular oxygen to produce ROS such as singlet oxygen (^1^O_2_), peroxide, or hydroxyl radical for the use in the killing of the cancer cells. The formation of radical oxygen species is often denoted as Type I and that of singlet oxygen is denoted as Type II PDT. The process, which is depicted schematically in Figure 2, is recognised as being minimally toxic. Many studies have been done regarding the currently used clinical procedure of PDT [23,24,25,26,27,28], and a well-recognized limitation of most existing photosensitizers is that they require excitation in the visible range (e.g., 600–700 nm) where absorption and scattering of the exciting light by tissues is relatively strong, and thus, an efficient treatment of deep lying tumors is not possible [21]. 

One avenue for the improvement in this area is the development of PSs that absorb in the near infrared (NIR), but another possibility to address this goal is to use the two-photon absorption (TPA) as first proposed by Bhawalkar et al. [30]. 

The use of TPA in PDT applications allows, indeed, for reduced photodamage of the healthy tissue surrounding the cancerous area when using an NIR excitation source in comparison to that operating in the UV-Vis range. These longer wavelengths (700–1100 nm) also allow for the treatment of deeper lying tumors. The depth of tumor that can be treated is dependent on the wavelength, which is determined by the photosensitizer that is used. Another factor is the illumination light dose that is used. For example, 0.75 mg kg^−1^ of redaporfin with a light dose of 10 J cm^−2^ (750 nm) leads to a necrosis depth of 2.5 mm [31]. Increasing the light dose to 25 J cm^−2^ leads to an improved necrosis depth of 4 mm. This was compared to the less potent Photofrin, where 10 mg kg^−1^ of Photofrin was administered with 90 J cm^−2^ (630 nm), leading to a necrosis depth of 3 mm. Preferential absorption within the beam focal region also helps in the fluorescence imaging of the area of interest because a high longitudinal image resolution is enabled through the use of TPA which, in effect, provides confocal imaging without the use of an aperture and with reduced interference from fluorescence light not created in the image plane [32].

However, there are several drawbacks to the use of TPA in PDT, one of them being the need for the use of relatively expensive laser sources, which are required to reach sufficiently high intensities (upon focusing the beam), typically in the order of GW/cm^2^ [33]. Apart from the progress in the technology of inexpensive femtosecond and picosecond laser sources, one avenue toward making TPA PDT more practical is through the development of specially designed photosensitizers that feature very high values of the TPA cross-sections. Much work has been done in the area of both the theoretical research and measurements of TPA spectra of newly developed sensitizers. The number of materials that can be used as reliable standards for the comparisons of TPA properties has also recently increased [1].

In addition to the efficiency of TPA, there are other factors that need to be considered when evaluating photosensitizers for TPA PDT, which are going to be discussed in more detail in the following sections, among which the most important are the singlet oxygen (or other ROS) quantum yield and the fluorescence quantum yield. ^1^O_2_, the form of molecular oxygen that has the total electron spin of zero, unlike the usual oxygen molecule in which there are two unpaired electron spins, being in the triplet state, is the main reactive species that causes the cytotoxicity desired for Type II PDT; the efficiency of its generation by a PS is quantified by the singlet oxygen quantum yield (Φ_Δ_). The process of ^1^O_2_ generation involves the intersystem crossing (ISC) of the PS [34]; the process is known to be enhanced by the heavy atom effect [35,36,37], for example where the oxygen atom in molecules has been replaced by sulfur or selenium [38,39,40]. The efficiency of ^1^O_2_ generation also depends on the environment the compounds are present in; common PSs, Foscan^®^, and protoporphyrin IX are known to aggregate in aqueous media, thus limiting their ^1^O_2_ generation capabilities [41,42]. However, Foscan^®^ has a reasonably high Φ_Δ_ of 0.65, whereas its fluorescence quantum yield (Φ_F_) is 0.07 [43]. This low-fluorescence yield is also influenced by the formation of aggregates owing to the hydrophobic nature of these types of PSs. The PSs with low Φ_F_ cannot be utilized for simultaneous therapy and imaging. However, they can be combined with other more fluorescent molecules to enhance their potential for use as theranostic (therapy + diagnostic) tools. 

There is a continued strong demand for the development of two-photon absorbing molecules in various applications; some well-known PSs are based on porphyrins or a related structure, such as chlorin and bacteriochlorin [44]. However, there are many other types of photosensitizers, and here, we will discuss a range of small and large molecules, which exhibit TPA, that can be used in PDT applications.

### 1.2. Evaluation of Parameters Relevant for the Use of Photosensitizers

As mentioned above, the development of photosensitizers for TPA PDT involves the evaluation of several parameters that are critical for a PSs application potential, the primary photophysical measurements being those of the TPA properties as well as those concerning the singlet oxygen production. We list here some of the experimental techniques and mention also an alternative approach of quantum chemistry-based estimation of TPA properties that can provide a useful guidance for the synthesis of new PSs.

Two-photon excited fluorescence (TPEF) [45,46]: The principle of this technique is that a sample, typically a solution of a TPA active and fluorescent dye, is exposed to the excitation by short laser pulses and the resulting luminescence is recorded and compared with the emission of a dye with well-known TPA spectrum. The technique is widely used for the determination of the TPA cross-section of photosensitizers. Its relative simplicity allows for rapid estimation of TPA properties of a number of candidate compounds. The factor that may be a difficulty here is that the technique essentially determines the so-called two-photon brightness, that is the product of the two-photon cross-section and the fluorescence quantum yield, i.e., σ_2_Φ_F_ [47]. This can be converted to the absolute two-photon cross-section value if the quantum yield of luminescence is assumed to be the same for TPA as that for one-photon excitation, which can be readily determined [48].

Z- scan [49,50,51,52]: The Z-scan technique is a direct way of recording nonlinear absorption, which is detected by moving the sample along the axis of a focused laser beam and observing changes in the transmittance, either of the whole beam (so-called open-aperture scan) or by probing only the central part of the beam with an aperture placed in the far field (closed-aperture scan) [49,53,54,55,56]. The advantage of the technique is that materials with low-fluorescence efficiency can be studied, since emission from the sample is not required, the data processing does not require calibration by nonlinear absorption standards (although relative measurements can be readily performed), and the closed-aperture version of the measurement is sensitive to both the nonlinear absorption and nonlinear refraction. However, the TPA cross-section values measured using the Z-scan method are sometimes exaggerated, since the technique does not distinguish between different mechanisms of nonlinear losses in the sample, and it includes also such effects as nonlinear scattering and excited state absorption. The measurements are also more tedious than in the case of TPEF. A modification of the technique called f-scan uses an electrically tuned variable focal length lens, which has the advantage of fast measurements and does not require the movement of the sample [22,57,58,59].

The TPA probability, as quantified by the value of the two-photon absorption cross-section σ_2_, can be related to the imaginary part of the cubic (third-order) hyperpolarizability of a molecule, which can be computed by quantum mechanics [60]; however, the computation of the dispersion of the hyperpolarizability to extract its imaginary part at the two-photon resonance would be extremely tedious. Instead, the properties are calculated at the two-photon resonance, and the two-photon spectrum is modeled by assuming a certain spectral shape of a given band. The expression for two-photon matrix elements (*S_αβ_*) for the absorption of photons with identical energies is shown in Equation (1) [61,62]:

(1) $$S_{\alpha \beta }=\underset{i}{\sum }\frac{\langle 0|\mu_{\alpha }|i\rangle \langle i|\mu_{\beta }|f\rangle }{\omega_{i}-\omega_{f}/2}+\frac{\langle 0|\mu_{\beta }|i\rangle \langle i|\mu_{\alpha }|f\rangle }{\omega_{i}-\omega_{f}/2}$$

*μ_α_* (or *μ_β_*) is the dipole moment operator (along a Cartesian direction, i.e., one of *x*, *y*, and *z* axes), ω*_i_* is the excitation energy of the intermediate state |i〉, and ω*_f_* is the excitation energy of the final excited state |f〉. 

The *TPA* absorption strength *δ_TPA_* is deduced via the averaging of *TPA* probability over orientation (for molecules in solution or gas phase) according to Equation (2) [61]:

(2) $$\delta_{TPA}=\underset{\alpha \beta }{\sum }\left[F\times S_{\alpha \alpha }S_{\beta \beta }^{*}+G\times S_{\alpha \beta }S_{\alpha \beta }^{*}+H\times S_{\alpha \beta }S_{\beta \alpha }^{*}\right]$$

where *F*, *G*, and *H* are factors related to the polarization of the light source (*F*, *G*, *H* = 2, 2, 2 for linearly polarized light and -2, 3, 3 for circularly polarized light, assuming the interaction of two identical photons). The *TPA* cross-section at the absorption band maximum can be computed with accounting for the factor describing the broadening of the band using Equation (3) [61,62], which allows for comparison to experimental measurements:

(3) $$\sigma_{2}=\frac{4\pi^{3}a_{0}^{5}\alpha }{c}\;\frac{\omega^{2}}{\Gamma_{ƒ}}\;\delta_{TPA}$$

where *a*_0_ is the Bohr radius, α is the fine structure constant, c is the speed of light, ω is the photon frequency, and Γ_ƒ_ is the broadening factor (typically chosen as 0.1 eV) [60,63,64].

Singlet oxygen detection: ^1^O_2_ detection can be performed in two ways. The first option is that of using direct detection, which is usually performed by steady-state or time-resolved phosphorescence measurements, with an emission detection at ≈1270 nm [65,66,67,68]. This can prove to be challenging in some cases, because ^1^O_2_ phosphorescence is very weak compared to the fluorescence of PSs, so it is reliant on the medium used for the experiment, the emission efficiency in water being particularly weak compared to other solvents. The presence of singlet oxygen can also be detected using indirect, chemical means, such as monitoring the oxidation of 9,10-diphenylanthracene via UV-Vis absorption spectroscopy at 372 nm [69,70,71], as well as the monitoring of N, N-dimethyl-4-nitrosoaniline bleaching at 440 nm [72,73,74]; these methods can be used to test for the presence of ^1^O_2_ in aqueous solutions. Electron spin resonance (ESR), or electron paramagnetic resonance (EPR), is a technique used in the study of molecules with unpaired electrons. ESR is a spin-trapping method to detect free radicals as well as ^1^O_2_ [75]. The direct observation of most free radicals is not possible due to them being highly reactive and having a short half-life; thus, they do not reach a measurable concentration. Indirect observation is done with ESR and the use of spin traps and spin-label reagents. A spin trap is a diamagnetic molecule used to stabilize the radical species by forming a spin adduct; this more stable species can reach measurable levels [76]. Some commonly used spin-label reagents for ^1^O_2_ trapping are 2,2,6,6-tetramethylpiperidine (TEMP) [77,78,79] and 2, 2, 6, 6-tetramethyl-4-piperidone (TMPD) [80,81,82]. For the indirect detection of ^1^O_2_, TEMP in the presence of ^1^O_2_ produces the stable nitroxide radical 2,2,6,6-tetramethyl-1-piperidinyloxyl (TEMPO), and ESR is able to detect the presence of this radical. This technique is suitable for organic solvents, such as DMF [83], as well as aqueous solutions [84,85].

## 2. Modifications of Photosensitizers for Improved Two-Photon Absorption 

There have been many studies concerning the design and optimization of the photosensitizer structures in order to enhance their TPA properties [86,87]. Modification of the molecular structure and performance of the PS has been performed in several ways including extension of the π-conjugated backbone [3,88], addition of a TPA antenna [89,90], metalation [91,92], encapsulation of the PS [7], and dimerization [93,94], amongst others. This discussion will begin with the modification of the conjugated π-system of the molecule. This can be accomplished in several ways, including modifying the symmetry of the molecule, its π-conjugated backbone, and the donor and acceptor behavior of the terminal and core groups.

Several requirements for maximizing the TPA cross-section of a chromophore are well known:

1. The presence of a long π-conjugated system with enforced co-planarity ensures large conjugation lengths to provide ease of delocalization of π-electrons;
2. Donor (D) and acceptor (A) groups located at the ends of the molecule and also possibly at its center (giving e.g., A-π-D or A-π-D-π-A or D-π-A-π-D structures where -π- denotes the conjugated linker) to provide dipolar or multipolar structures (quadrupolar, octupolar etc.) and large transition dipole moments;
3. Narrow one-photon and two-photon absorption bands.

In general, increasing the strength of the donors and acceptors, whether symmetrically or asymmetrically, may lead to the enhancement of the TPA cross-section values. The TPA cross-section may also be significantly enhanced by increasing the spatial extent of the π-conjugation, which leads to the increase in the delocalization of the electrons [61]. The planarity and rigidity of a molecule also seem to be critical in obtaining high TPA cross-section values [95]. 

One should distinguish between the cases of asymmetric molecules (in the simplest case of the A-π-D type), thus possessing a non-zero dipole moment, both in the ground state and in its excited states and centrosymmetric molecules where the dipole moment is null. The fundamental difference between these two types of two-photon chromophores is in the selection rules applying to the absorption transition [3]: for the non-centrosymmetric ones, the excited states (S_1_, S_2_ etc.) are accessible by both one-photon and two-photon transitions (note that in naming the states S_n_, we assume that the molecules possess a singlet ground state, which is true for most organics), while the centrosymmetric systems allow only one-photon transitions between states of different parity (*gerade*↔*ungerade*) and two-photon transitions between states of the same parity (*gerade*↔*gerade* or *ungerade*↔*ungerade*). Quite often, the lowest lying one-photon transition (e.g., from ^1^A_g_ ↔ ^1^B_u_) is not two-photon allowed, while the transition from ^1^A_g_ ↔ ^2^A_g_, which may be at a higher energy, is one-photon forbidden but two-photon allowed. 

In the case of non-centrosymmetric molecules, absorption may result in a large change of the dipole moment. In fact, for charge-transfer molecules, the sign of the dipole moment may even be inverted. For centrosymmetric systems, on the other hand, the electronic excitation may be accompanied by a considerable charge transfer from the end groups to the central linkages; this results in a large change in the quadrupole moment. Symmetrically substituted molecules that show this behavior may have quite large TPA cross-section values [96]. However, asymmetrically substituted molecules can also compete with the increased TPA cross-section values, as described in more detail in the following sections [97,98].

An important thing to note is that the TPA cross-sections measured in non-polar solvents are usually larger than the values for the same compounds determined in more polar solvents. This is due to a solvatochromic effect, which is a complex phenomenon as described in more detail by Reinhardt et al. [95] and discussed for the two-photon absorption case by Wielgus et al. [99,100,101,102,103].

## 3. Two-Photon Absorption Molecules: Small Molecules

**Figure 3:**
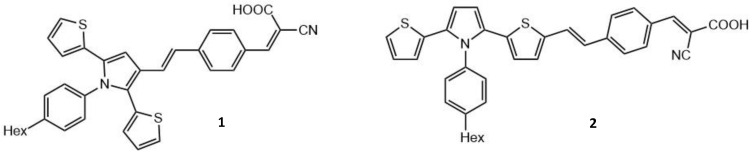
Structure of **1** and **2**.

Surprisingly, some relatively small organic molecules may be interesting compounds for TPA-based applications in therapy and diagnostics. One such example is 2,5-dithienylpyrrole (DTP), which is an excellent electron-donating compound, used by Sharmoukh et al. They demonstrated that the modification of DTP could improve its TPA efficiency [104]. Two DTP-based dyes, **1** and **2** (see Figure 3), were designed and synthesized, and their nonlinear absorption properties and photophysical profiles and behaviors were assessed [105].

The D-π-A structure of **1** and **2** [104,106] allows the presence of charge-transfer electronic excited states. It has been shown that **1** and **2** have large TPA cross-sections, especially for the S_0_→S_2_ transition (between 500 and 700 nm) with values of 1300 and 8000 GM, respectively. The result for **2** is among the highest TPA cross-sections found in the literature for small molecule compounds [3]. The S_0_→S_1_ TPA transitions were seen in the 700–1000 nm range, with cross-section values of 400 and 1000 GM for **1** and **2**, respectively. These values have been related to the presence of the conjugated thiophene rings, similar to boron-containing arenes that were previously studied by some of the same authors [107]. The larger cross-section values are attributed to the thiophene ring being covalently linked to the π-bridge, as seen in **2**. In addition, in **2**, the phenyl group is almost orthogonal to the rest of the chromophore and does not participate in the π-conjugated backbone.

### 3.1. Highly Branched Molecules

**Figure 4:**
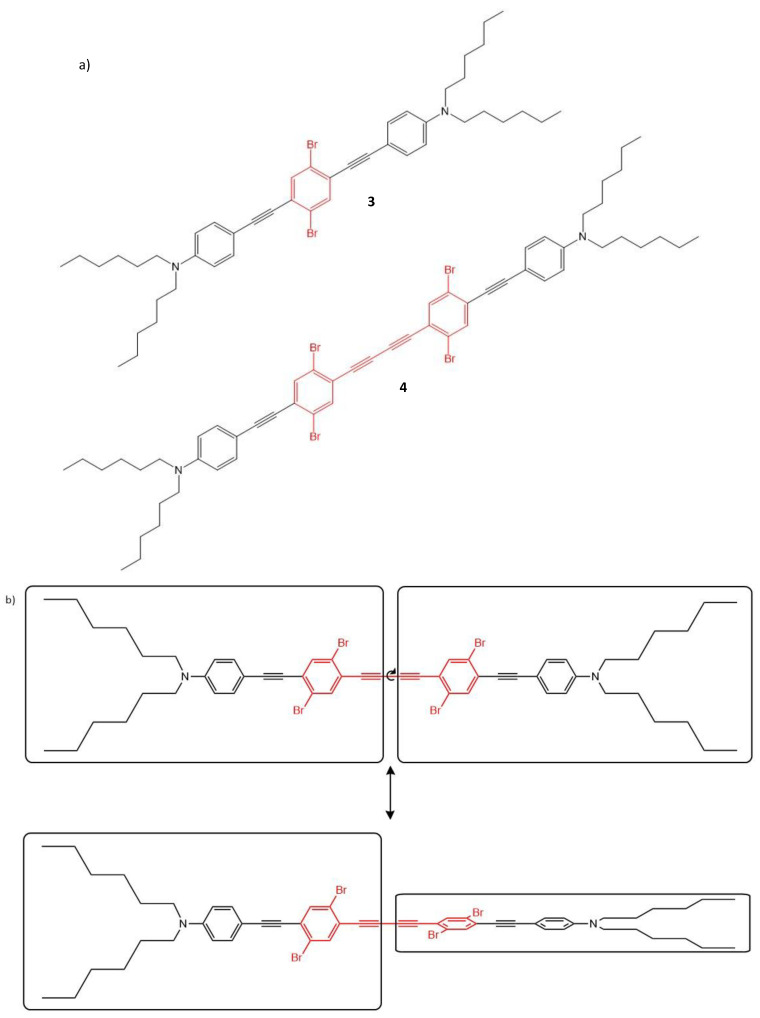
(**a**) Structure of the centrosymmetric (**3**) and non-centrosymmetric (**4**) chromophores. (**b**) Chromophore **4** in its excited state (**top**) has D_2h_ symmetry, and in the ground state, (**bottom**) it has D_2d_ symmetry.

Chromophores featuring dibromobenzene moiety and diyne bridges (chromophores **3** and **4**, Figure 4) have been studied [108]. The long terminal alkyl chains of chromophore **3** and **4** reduce aggregation and improve their solubility. Even though both structures look centrosymmetric, chromophore **4** was found to exhibit a non-centrosymmetric behavior, which is due to the non-planar conformation it adopts in its ground state (shown in Figure 4b), resulting in its “broken symmetry”.

From theoretical considerations, it is known that the sum-over-state terms including the difference between the dipole moments of the ground state and the excited state contribute to the TPA cross-section of non-centrosymmetric molecules, the contribution being absent in centrosymmetric molecules [97]. The effect of breaking the symmetry, due to the addition of the second diyne bridge and second dibromobenzene moiety in **4**, causes the red shift of TPA spectrum observed for chromophore **4**, in comparison to that of **3**, with the TPA cross-sections at 800 nm being ca. 200 GM and 31 GM (800 nm) for **3** and **4**, respectively. 

### 3.2. Zwitterion Molecules

**Figure 5:**
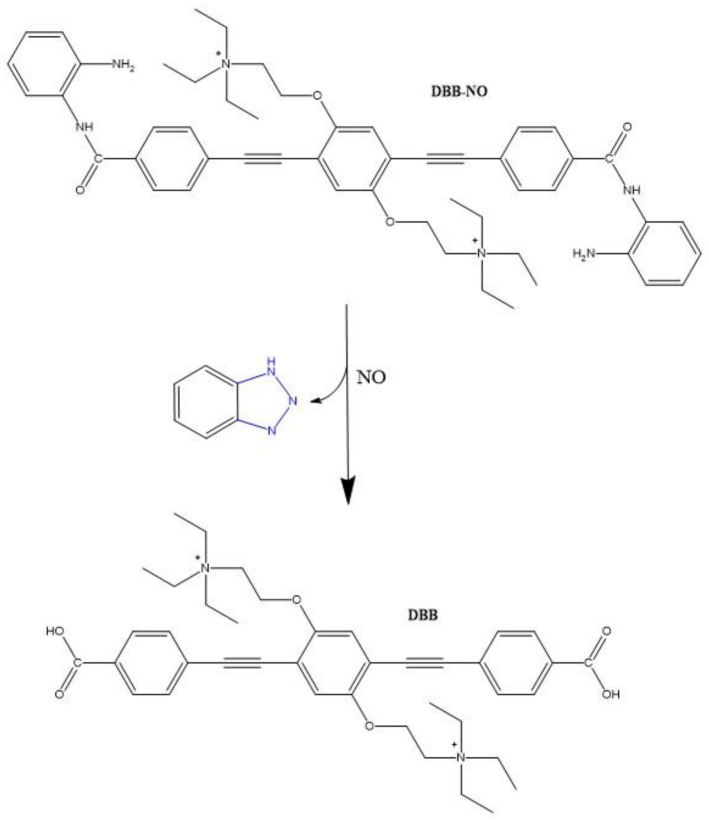
Molecular structure of the two-photon absorbing fluorescent PS (TP-FPS) **5** (**top**) and after its interaction with NO to form the zwitterionic structure **6** (**bottom**).

In an interesting study, Hu et al. described a nitric oxide (NO) activatable fluorescent photosensitizer, **5** (Figure 5) [109]. When **5** interacts with NO, the *o*-phenylenediamine moiety is removed, leaving a more fluorescent molecule (**6**). Compound **6** was shown to have an increased TPA cross-section value of 2800 GM (710 nm) compared to a value of 270 GM (710 nm) for **5**. It was reported that the formation of the zwitterion structure of **6** was responsible for this improved TPA cross-section value. The generation of ROS was monitored chemically following the degradation of anthracene-9,10-diyl-bis-methylmalonat. The results determined the singlet oxygen quantum yield (Φ_Δ_) of **6** to be 89%, while only 1.2% was obtained for **5**. The fluorescence quantum yield (Φ_F_) was also calculated to be higher for **6** compared to **5**, 9.3% and 1.2%, respectively. These results display great potential for these molecules to be used for both NO-activatable two-photon imaging and two-photon PDT. 

**Figure 6:**
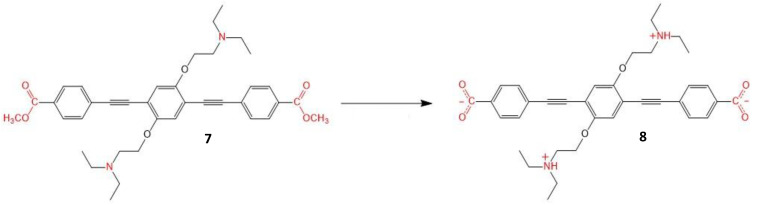
Structure of the precursor (**7**) and the inner-salt-like TPA-PS (**8**).

Another interesting example of a small molecular photosensitizer is the inner-salt structure (**8**) designed by Hu et al. (Figure 6). This molecule contains two phenyl-acetylene units, which is a frequently used backbone in the design of two-photon absorbing compounds, acting as the main π-conjugated linker between the two end donor groups and the central acceptor moiety [110]. Additional bridging leads to an increase in the conjugation length, thus increasing the distance over which the charge inside the molecule can be transferred, which is a key point of these structures. This, together with the increase in the extent of symmetrical charge transfer from the donor ends of the molecule to the acceptor moiety in the middle, results in a large increase TPA cross-section, to 4857.4 GM (720 nm, methanol), and the shift of the absorption maxima to shorter wavelengths (λ = 309 nm and 356 nm compared to λ = 320 nm and 381 nm of its precursor, **7**).

### 3.3. Small Molecule-Based Salts

**Figure 7:**
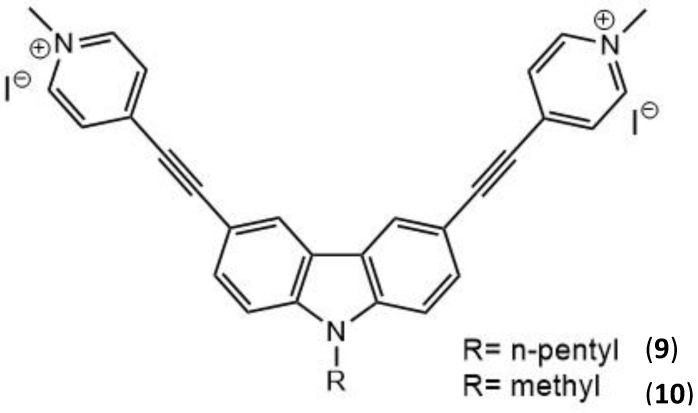
Structures of **9** and **10**.

Carbazole-based cyanines were found to be suitable as TPEF probes for both DNA and cell imaging due to their high binding affinity to DNA; they also possess large TPA cross-sections and good water solubility [111,112]. For example, two carbazole derivatives, **9** and **10**, whose structures are displayed in Figure 7, were designed and synthesized by Zheng et al. [113].

These molecules display a C_2v_ symmetric A-π-D-π-A structure, with strong intramolecular charge transfer and planarity, with positive charges. All these factors contribute to the large two-photon absorption cross-section, the low fluorescence quantum yield and the high binding affinity toward DNA by the intercalation mode [114,115,116]. The molecules show ability for the efficient photocleavage of DNA, which may be excited not only by visible light but also by 800 nm NIR under both aerobic or anaerobic condition via a type I mechanism. The ionic groups act as the electron acceptor groups of the molecule and are introduced by salification (conversion to a salt) reaction. The two-photon absorption cross-sections were determined, and their values at 760 nm were 522 GM and 492 GM for **9** and **10**, respectively. The high binding affinity to DNA of these molecules is due to their symmetric bis-cationic and planar structures, implying the potential for them to be DNA photocleavers. The experimental evidence indicated that DNA photocleavage occurs mainly via the abstraction of hydrogen by N-centered radicals (type I mechanism), contributing to the DNA photocleavage abilities in anaerobic conditions. These carbazole-based photocleavers gave valuable results for the further development of new two-photon excited PDT agents.

Following on from the above-mentioned study, the same two carbazole-based ethynylpyridine salts, **9** and **10**, were further utilized as photosensitizers for antibacterial studies. Their antibacterial activity was tested against *Escherichia coli* (*E. coli*) [117]. An antibacterial mechanism was proposed based on electron paramagnetic resonance characterization, which indicated that a nitride radical is generated upon laser irradiation. This would allow the potential for further applications of **9** and **10** in PDT. Carbazole compound families have been known to have a variety of properties including anticancer, antibacterial, anti-HIV, and anti-inflammatory [118]. Their affinity toward DNA allows the drugs to bind more easily with bacterial DNA, displaying their potency against bacteria [112]. Carbazole-based derivatives have a large conjugated system that leads to a large TPA cross-section and contributes to the large intramolecular electron transfer, thus helping to stabilize the cation that is formed [119]. These cations that are formed by the carbazole-based derivatives have been known to demonstrate high photochemical stability, unique biological activity, and photoelectronic properties which are expected to be of greater importance against the inhibition of Gram-negative bacteria [118,120,121]. The fluorescence emission spectra for **9** and **10** were measured with an excitation wavelength of 425 nm in phosphate-buffered saline (PBS) solution. They exhibited fluorescence emission peaks at 576 and 592 nm for **9** and **10**, respectively. Their fluorescence quantum yields were also calculated using fluorescein as a reference standard (Φ_F_ = 0.9) in PBS buffer solution, to be 2.0 × 10^−4^ and 6.0 × 10^−5^ for **9** and **10**, respectively [122]. These quantum yields are so low due to the fluorescence quenching that occurs because of the intramolecular D–A electron transfer [123,124]. Results from this study showed that almost no *E. coli* cells can survive in the presence of **10** under laser irradiation (442 nm, 20 mW/cm^2^) for 10 minutes. When **9** is used, *E. coli* was shown to be inhibited after only 30 seconds of laser irradiation; this improved photoinduced antibacterial activity is mostly attributed to the strong one-photon absorption and the cationic radicals that are formed under laser irradiation.

### 3.4. Organometallic Molecules

**Figure 8:**
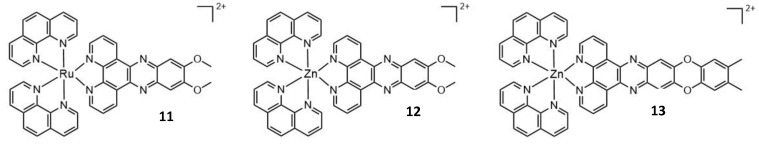
Structure of **11**, **12**, and **13**.

Due to their remarkable photophysical properties, transition metal complexes can be viewed as attractive PS candidates [6,92,125]. In particular, ruthenium (II) polypyridyl complexes have gained widespread attention over the years due to their evident advantages, including large TPA cross-section, long triplet lifetime, high chemical stability, photostability, and high ^1^O_2_ yields. In addition, their properties can also be easily tuned [91]. Ruthenium is a popular choice for metalation as it has low toxicity, its compounds can have good water solubility, and it is rapidly excreted in vitro. However, ruthenium is a metal with a high price tag; hence, the more common metal choices are also explored, e.g., zinc. The design of two [Zn(phen)_2_ dppz]^2+^ derivatives for two-photon PDT, as well as the photophysical properties of [Ru(phen)_2_ dppz]^2+^, evaluated by the time-dependent density functional theory were reported [126]. Recent work by Hess et al. demonstrated that the [Ru(phen)_2_ dppz]^2+^ derivative (**11**, Figure 8), with the modification of having two OMe groups on the dppz ligand, could successfully be used as a PS for two-photon PDT [127].

The complex demonstrated strong anticancer activity, high ^1^O_2_ quantum yields, efficient cellular uptake, and a large TPA cross-section (σ_2_ = 245 GM, 800 nm) [126]. The photophysical properties of **11** and **2**, which were relatively inexpensive because of the use of zinc instead of ruthenium [Zn(phen)_2_ dppz]^2+^ complexes (**12** and **13**, Figure 8), were measured. For **12**, the central ruthenium in **11** is replaced with zinc, for **13**, the π-conjugated backbone is further extended compared to the first two complexes. For compounds **11–13**, it has been shown that changing the central metal has a great effect on the TPA properties. It has already been mentioned that in order to produce high ^1^O_2_ quantum yields, the PS needs to have sufficiently high triplet (T_1_) energy that is larger than that of singlet oxygen (0.98 eV) [128,129]. The T_1_ energy values were determined to be 2.13 eV for **11**, 2.13 eV for **12**, and 2.00 eV for **13**. In reference to spin-orbit coupling, the heavy atoms increase the ISC because the SOC effect increases with the atomic weight. Thus, the heavy atom effect of Ru is larger than that of Zn. Nevertheless, complex **11** showed efficient production of ^1^O_2_ (Φ_Δ_ = 0.75), and the T_1_ states of **12** and **13** are higher than 0.98 eV, making sure they can also effectively produce singlet oxygen. They all demonstrate the potential to be used as PSs for two-photon PDT application. In particular, **13** has demonstrated an improved TPA cross-section through extended π-conjugation (σ_2_ = 98.9 GM, 705 nm) compared to **12** (σ_2_ = 10.58 GM, 705 nm), and it comes at a reduced cost compared to the ruthenium containing **11**.

## 4. Two-Photon Absorption Molecules: Large Molecules

### 4.1. Dimers

**Figure 9:**
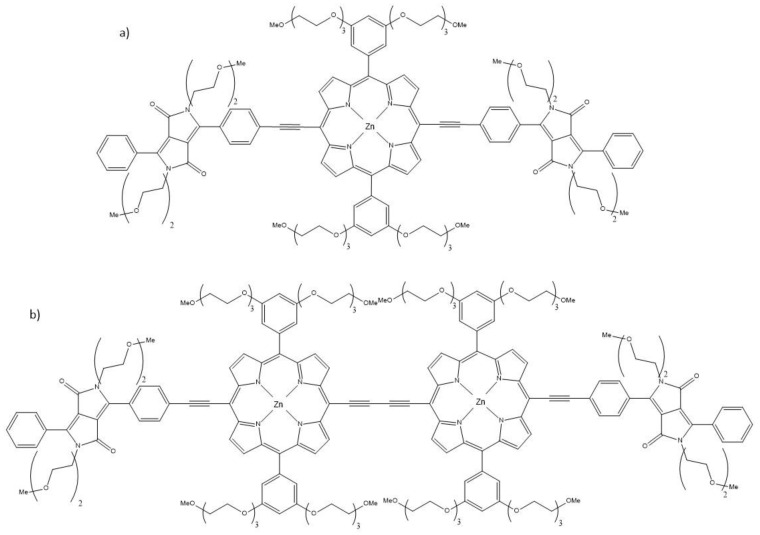
(**a**) Structure of diketopyrrolopyrrole–zinc porphyrin conjugate (**14**) and (**b**) the π-extended porphyrin dimer (**15**).

Porphyrin-based compounds, and various tetrapyrrolic-based compounds, have found widespread application as PSs for PDT. In this review, we survey the modifications that have been introduced to improve their TPA capabilities needed for two-photon-based PDT. One strategy of increasing the π-conjugation and therefore, the TPA properties of the compound is simply to have additional functional groups attached to the core molecule such as a macrocycle. An example of the use of that strategy is the diketopyrrolopyrrole–metalloporphyrin conjugate (**14**), the structure of which can be seen in Figure 9a [125]. To further expand upon molecule **14**, its Zn-porphyrin dimer can also be utilized with the further extended π-conjugation via the addition of the two diketopyrrolopyrrole (DPP) moieties, 5-(4-diphenylaminostilbene),15-(2,6-dichlorophenyl)-21H,23H-porphine (**15**) (Figure 9b) [130]. 

The extension of the porphyrin π-conjugated aromatic system is an effective method to decrease the HOMO-LUMO gap for the development of NIR absorbing and emitting materials. The dimer species shown in Figure 9b features splitting of the Soret absorption band (ca. 460 nm and 505 nm), which is indicative of strong electronic interactions between the two porphyrins. A bathochromic shift observed in the absorption spectra for **15** is ascribed to the enhanced conjugation of the molecule. With an increase in the conjugation length and the introduction of the electro-active DPP system on either side of the porphyrin dimer, a strong increase in the two-photon absorption cross-section value is observed to 21,500 GM at 910 nm compared to 4000 GM for the monomer. The TPA maximum is also red-shifted, but there is a decrease in the singlet oxygen quantum yield (0.19 for the dimer species, compared to 0.38 for the monomer species), which can be attributed to the lowering of the triplet energy level (ca. 1.0 eV compared to 1.80 eV for the monomer) due to the extended conjugation of the dimer [131]. Due to the lower energy of the triplet state, because of the closeness of the triplet energy level to that of singlet oxygen (0.98 eV), there is less efficient triplet–triplet energy transfer; thus, a lower singlet oxygen quantum yield is observed for the dimer **15**. However, with the large TPA cross-section value of 21,500 GM, the conjugated dimer can still be considered as a promising candidate for imaging applications.

**Figure 10:**
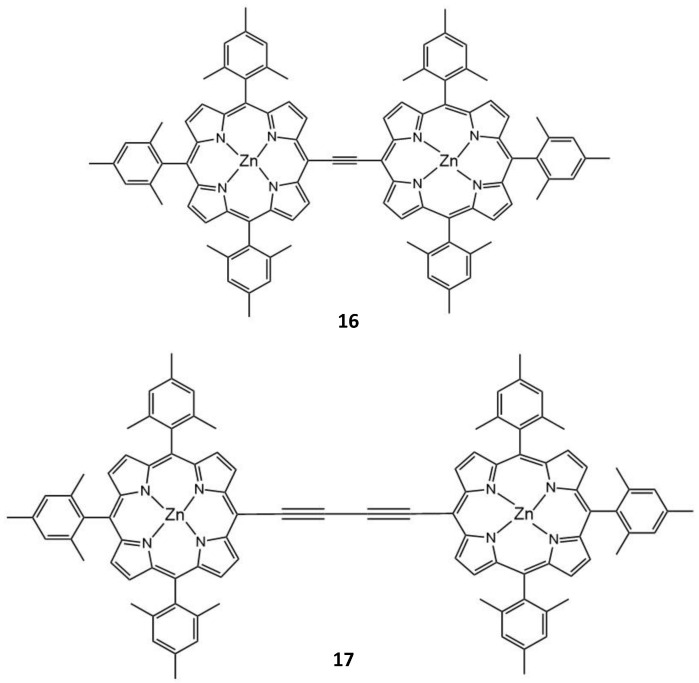
Structures of **16** (ethyne linked) and **17** (di-ethyne linked) Zn porphyrins.

Another example of the conjugated linkage of zinc porphyrins to form dimers for TPA and ^1^O_2_ generation was discussed by Mazur et al. [22]. ^1^O_2_ yield was determined for the dimers **16** and **17** (Figure 10) to be Φ_Δ_ = 0.47 and 0.27, respectively. The TPA cross-section values of 3115 GM and 3700 GM (725 nm and 775 nm), respectively, show that especially the dimer **17,** absorbing at longer wavelengths, can be promising for the treatment of deeper lying tumors.

Another effect, which can be utilized to improve the TPA properties of the porphyrins is that of metalation. However, this can have both positive and negative effects. The metal ion in the porphyrin usually leads to a decrease in Φ_Δ_ but an increase in σ_2_, so there can be a trade-off between the two factors in the influence on the TPA PDT suitability (which depends on the σ_2_Φ_Δ_ product) of the compounds [22]. A reason for this observed decrease in φ_Δ_ values is an increase in the back intersystem crossing (ISC) rate to the ground state [132]. Compounds with a torsional angle of 0°, planar molecules, such as the dimers linked by ethyne (**16**) and di-ethyne (**17**), show an enhanced TPA cross-section and ^1^O_2_ generation, as well as faster ISC compared to twisted conformers. The large π-delocalization of dimers **16** and **17** leads to Φ_Δ_ = 0.27–0.47. The maximum value of σ_2_ measured at 725 nm was for dimer **16** (8200 GM) compared to dimer **17** (5500 GM). There was a strong enhancement of the singlet oxygen quantum yields observed for dimer **17.** Two-photon excited singlet oxygen generation was found to be 500 times more efficient than that for tetraphenyl porphyrin alone [133], which is due to an increase in the conjugation length, where the π-delocalization is maximized in the large planar structure.

### 4.2. Dendrimers

**Figure 11:**
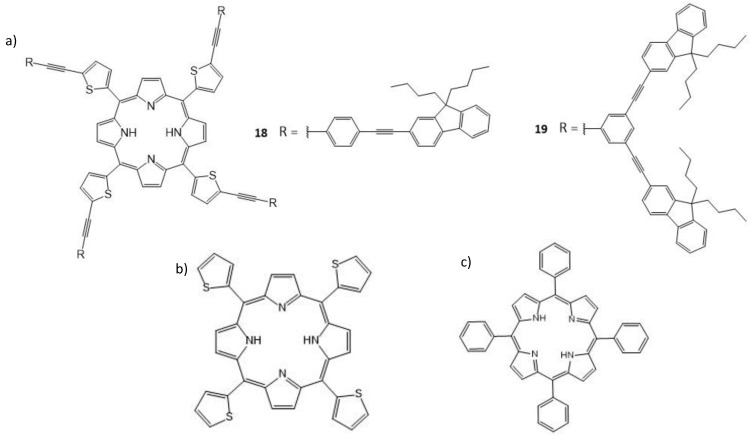
(**a**) Structures of dendrimers **18** and **19**. (**b**) Structure of TThP and (**c**) Structure of TPP.

Another interesting study by Kim and Cho deals with a series of conjugated meso-porphyrin dendrimers containing conjugated fluorenyl dendrons (Figure 11a), which is based on a central meso-tetra(thien-2-yl) porphyrin core (TThP, Figure 11b) [134].

Dendrimers **18** and **19** were able to efficiently photogenerate singlet oxygen; they also turned out to be fairly good two-photon absorbers, showing their potential as TPA-based photosensitizers. In that respect, the advantage of the thienylene linkers over the phenylene linkers on the TPA cross-sections is clearly evidenced. Indeed, the *meso*-2,5-thienylene groups within the given conjugated π-manifold (while preserving its overall symmetry) resulted in a significant improvement of the TPA cross-section (σ_2_) compared to TThP (13 GM). The Φ_F_ values of analogues **18** and **19** were 0.04 and 0.025, respectively, as determined by using TThP as a reference (Φ_F_ = 0.05). Energy transfer (ET) between the peripheral dendrons and the porphyrin core was probed, **18** and **19** being excited at the dendron absorption wavelength of ca. 320 nm. Dendrimer **18** displays exclusively red emission, which reveals efficient ET from the dendrons to the core. Dendrimer **19** also shows strong red emission, but it also exhibits a broad, weak residual band at ca. 375 nm. This suggests that ET from the dendrons to the porphyrin core is less efficient in **19**, indicating that higher generation dendrons are less effective in transferring their energy in spite of their larger linear absorption. TPEF of the dendrimers was measured in dichloromethane. The results showed a quadratic dependence of fluorescence intensity on the excitation power at all wavelengths of the spectra, as expected for TPA, and the maximum cross-sections were 730 GM for dendrimer **18** and 580 GM for **19** (λ_ex_ = 800 nm). The singlet oxygen quantum yields were determined to be 0.73 and 0.50 for **18** and **19**, respectively. The Soret band of **18** (442 nm) and **19** (438 nm) is red-shifted (compared to the Soret band of TThP (425 nm)), which was recognized as being indicative of better π-conjugation of the peripheral dendrons with the central porphyrin core. The decrease in Φ_F_ (**18** = 0.04, **19** = 0.025 compared to tetraphenylporphyrin (TPP, Figure 11c) = 0.11, all in toluene) can be related to the presence of the S atom, which may possibly favor a non-radiative decay process via spin–orbit coupling. This study reinforces the idea that improving the conjugation, in this case between the porphyrin core and the *meso*-substituents, is a key factor in improving the TPA potential of a molecule. Here, the 2,5-thienylene spacer allows for better conjugation. The dendrimers in this study displayed improvements in σ_2_ values, while still maintaining sufficient fluorescence for measuring TPA cross-sections by TPEF.

### 4.3. BODIPYs

There is also much interest in BODIPY (4,4-difluoro-4-bora-3a,4a-diaza-s-indacene, the abbreviation comes from the common name boron-dipyrromethane)-based compounds for various applications related to their favorable optical properties such as high fluorescence quantum yields, narrow emission bands and small Stokes shifts; however, the unmodified BODIPY structure molecule, which has long been used in bioimaging and as a chemo-sensor [135,136,137,138,139,140,141,142], has a relatively low two-photon absorption cross-section, about 50 GM, which limits its nonlinear optical applications. 

**Figure 12:**
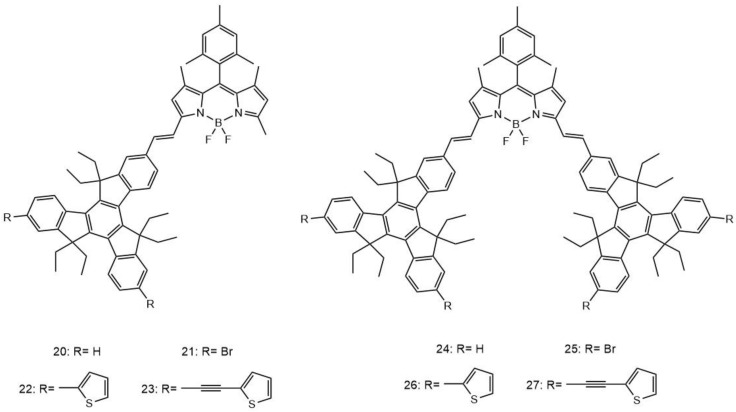
Structures of the BODIPY derivatives **20**–**27**.

There has been recent interest in the modification of BODIPY dyes with truxene derivatives [143]. The truxene molecule is a C_3_-symmetric aromatic structure with high delocalization of the π-electrons. These truxene molecules also exhibit good co-planarity and thermal stability. In a recent study, eight green and blue BODIPY dyes substituted by a range of truxene derivatives were characterized (Figure 12) [144].

Different functional groups including bromine, thiophene, and ethynylthiophene were introduced at the 7, 12-positions of the truxene moiety in order to further extend the π-conjugation and thus improve the TPA properties of the resulting structures. As mentioned previously, several different strategies can be utilized in order to enhance the TPA cross-section of compounds, such as extending the π-conjugation backbone [145], the introduction of strong electron acceptor and/or donor groups at the center or at the terminal parts of the molecule [146,147], increasing the amount of branching of the molecule [148,149], and (or) improving the co-planarity of the molecules [150]. The unsubstituted compounds **20** and **24** displayed the characteristic absorption band of the truxene core at 307 nm, while for the bromine-substituted compounds **21** and **25**, it was 313 nm and 307 nm, respectively. A red-shift to 340 nm is observed with the addition of thiophene, for compounds **22** and compound **26**, and to 341 nm with the addition of ethynylthiophene, both **23** and **27**, which can be explained by the extension of the π-conjugation between the truxene unit and the thiophene and ethynylthiophene moieties. The mono-truxene substituted BODIPYs (dyads, compounds **20**–**23**) displayed higher fluorescence quantum yields, in the range of 0.55–0.63, compared to those displayed by the di-truxene substituted BODIPYs (triads, compounds **24**–**27**), with fluorescence quantum yields measured to be 0.36–0.52. These lower observed fluorescence quantum yields could be due to the increase in internal conversion between the narrower band gaps of the triad truxene BODIPYs [151,152]. There was no heavy atom effect observed for compound **21** and compound **25**, which was most likely due to the long distance between the BODIPY species and the Br atoms [144,153]. The σ_2_ values were calculated to be 1000 GM, 880 GM, 1100 GM, 750 GM, 1600 GM, 1750 GM, 1100 GM, and 980 GM for **20**–**27**, respectively. Between the one- vs. two-truxene units, no significant impact was observed on their TPA cross-section values. 

**Figure 13:**
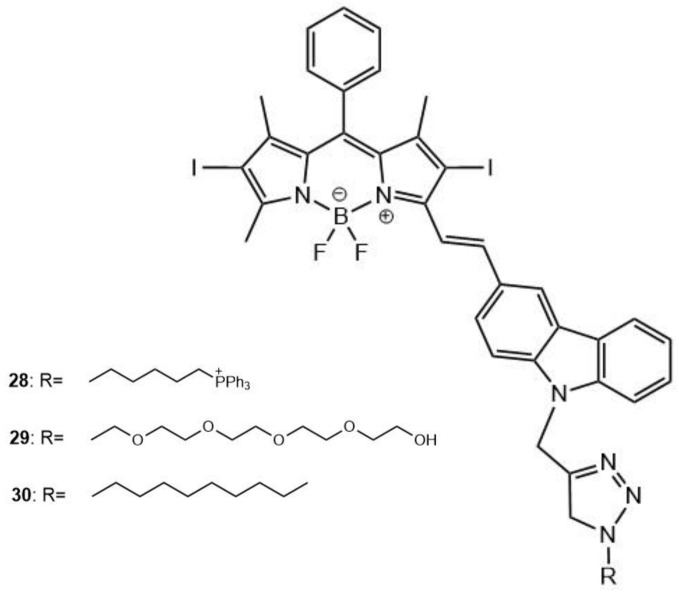
Structures of the BODIPY-based dyes **28**, **29**, and **30**.

Another BODIPY-based dye containing iodine substituents, **28** (Figure 13), was studied as a possible cationic TPA photosensitizer that could efficiently target tumor cells [154]. Two neutral molecules were also synthesized (**29** and **30**) for comparison to the cationic derivative.

It has been shown that the cationic group of **28** better improves solubility and cellular uptake, with structure-inherent targeting in vivo, and also inducing early apoptosis. It is known that dyes containing a cationic group usually show subcellular localization in the mitochondria; this is due to the high affinity of the cationic group toward the negative potential of the mitochondrial membrane [155,156]. The TPA of **28** was tested in PBS using the TPEF method with rhodamine B as a reference; the TPA maximum was observed at 808 nm, and the cross-section was calculated to be 25.5 GM. The addition of iodine in **28** promotes the ISC process of S_1_ → T_1_ transition, leading to more efficient and greater generation of ROS, including ^1^O_2_. The ^1^O_2_ production was followed chemically using 1,3-diphenylisobenzofuran (DPBF) as a scavenger, in dichloromethane. The singlet oxygen quantum yields (Φ_Δ_) were determined, using methylene blue as reference, to be 0.39, 0.31, and 0.33 for **28**, **29**, and **30**, respectively. These results confirm the potential for **28**, in particular, to be used in deep tissue tumor therapy [154,157] as well as for imaging-mediated PDT [137,138,139].

### 4.4. FRET Systems

**Figure 14:**
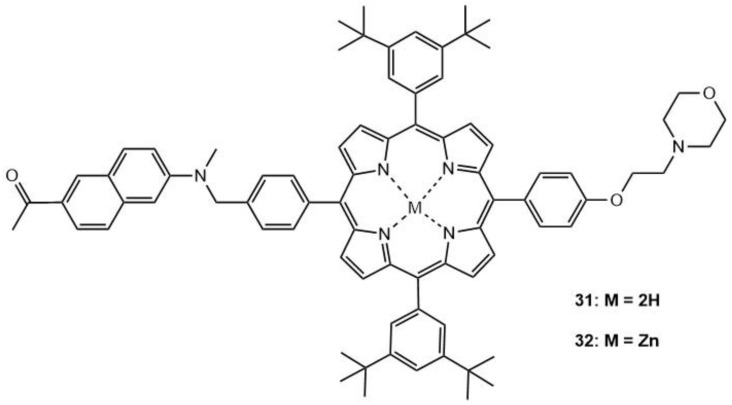
Structure of two dyads; **31** and **32**.

An example of the effects of metalation, as underlined previously, was reported in a study that explored the development of two different fluorescence (Förster) resonant energy transfer (FRET) dyads for two-photon excitation PDT (TPE-PDT) and fluorescence imaging [158]. In addition to the factors that relate directly to those modalities, cellular uptake efficacy and subcellular localization should be considered for the development of effective imaging and PDT agents. A TPA donor and lysosome targeted moiety were attached to a porphyrin acceptor, one being a free base (**31**) and the other being a Zn-metallated porphyrin (**32**); their structures are shown in Figure 14. The AceDAN donor (2-acetyl-6-dimethyl amino naphthalene), upon irradiation (using 740 nm NIR light), transfers its excited state energy to the porphyrin acceptor via the FRET mechanism.

AceDAN is a typical two-photon excitable chromophore. The morpholine moiety is included for lysosome-targeted cell imaging. 3,5-di-tert-butylphenyl groups on the porphyrin are expected to suppress the tendency of aggregation; this could increase the solubility of the compounds, being beneficial for fluorescence emission and ^1^O_2_ generation. **31** has a TPA cross-section of 112 GM, and **32** has a slightly smaller cross-section of 95 GM (λ_ex_ = 740 nm); these large TPA cross-sections are inherited from the AceDAN moiety (without AceDAN, the TPA cross-section values of the porphyrins are 25 and 11 GM, respectively). The TPA of the AceDAN donor can be utilized to efficiently generate the excited states of the porphyrin acceptor via FRET. To evaluate the potential of ^1^O_2_ generation, a chemical trapping method was utilized with DPBF as an ^1^O_2_ scavenger. The presence of **31** and **32** leads to a fast decay of DPBF upon irradiation (**31** > **32**). Upon one- or two-photon excitation of the AceDAN donor, intramolecular FRET processes occur with high energy transfer efficiencies (η_FRET_ = 97–98%), leading to formation of the excited porphyrin acceptor, from which deep red fluorescence and ^1^O_2_ can be generated and utilized for simultaneous cell imaging and PDT. The generation of ^1^O_2_ involves ISC of the excited porphyrin moiety and intermolecular triplet-triplet energy transfer to ground-state ^3^O_2_. NIR phosphorescence of ^1^O_2_ data shows Φ_Δ_ = 0.57 and 0.66 for **31** and **32**, respectively. Therefore, **32** shows increased ^1^O_2_ Φ_Δ_; this is attributed to the different distribution of S_1_ and T_1_ states between the metalated and non-metalated porphyrins. Strong red fluorescence of **31** and **32** was employed for TPEF cell imaging experiments under irradiation with a 740 nm femtosecond laser for tracking the significant morphology changes of A549 cells. 

**Figure 15:**
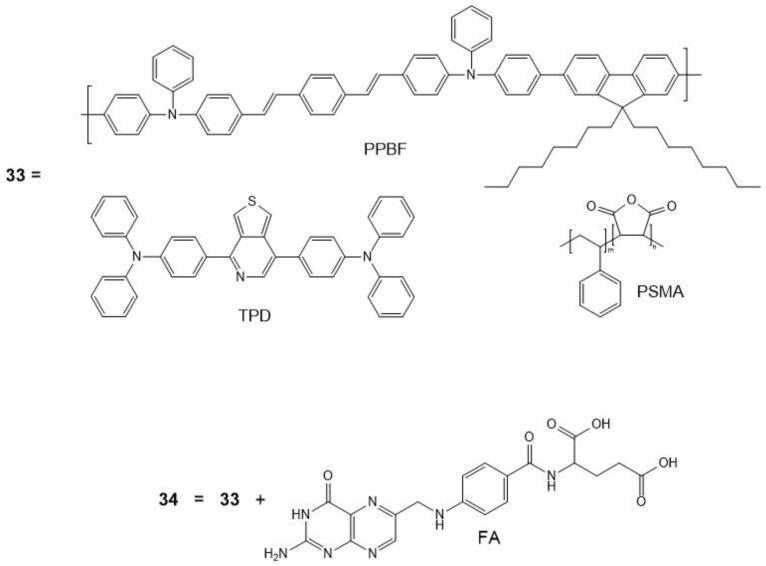
Structure of the components of the polymer-based nanoparticles **33** and **34**.

In general, the combination of a PS and a red-emitting dye is an interesting avenue for the combined imaging and PDT application. Multifunctional polymer-based nanoparticles that consist of a conjugated polymer (PPBF), a two-photon absorbing PS (TPP), and a red-emitting dye (4,4′-([1,2,5]thiadiazolo[3,4-c]pyridine-4,7-diyl)bis(N,N-diphenylaniline), TPD), and co-polymer poly (styrene-*co*-maleic anhydride) (PSMA) were studied by Duan et al. (Figure 15) [159].

Functionalization of the photosensitizers with folic acid (FA) (**34**) groups allows for the selective targeting of cancer cells. PPBF acts as the donor moiety with TPP and TPD each acting as an acceptor. PPBF has a large two-photon absorption cross-section of 529 GM per repeat unit (λ = 750 nm) and a high fluorescence quantum yield (ca. 0.84 in tetrahydrofuran (THF)). TPP has been extensively studied and used for such applications; here, it was chosen for its high singlet oxygen generation capability (yield of ca. 0.63) [160]. The red-emitting component, TPD, is known for its imaging contrast properties and its high fluorescence quantum yield of ca. 0.44 (in hexane) [161]. Efficient energy transfer for this particular combination is assured by the emission spectrum of PPBF overlapping reasonably well with the corresponding absorption spectra of TPP and TPD. The inclusion of poly (styrene-*co*-maleic anhydride), PSMA, a non-emitting co-polymer, provides hydrophilicity to the nanophotosensitizer, improves the stability through the carbonyls of PSMA, and suppresses the self-quenching of PPBF. The two-photon absorption cross-section was measured for the nanoparticles **34** to be ca. 8.6 × 10^6^ GM (λ = 750 nm), which is a significant increase when compared to bare TPP and TPD, 12 GM and 97 GM, respectively. The singlet oxygen production of nanophotosensitizers **33** was determined photochemically by following the oxidation of 9,10-anthracenediyl-bis(methylene)dimalonic acid. The combined PPBF/TPP/TPD/PSMA structure shows enhancement of the TPP and TPD two-photon emission by ca. 161 and ca. 23 times, respectively. Two-photon excited fluorescence signals from cancer cells incubated with **34** and TPD-FA were compared; strong emission was observed with those incubated with **34**, whereas almost no florescence was observed from the cells incubated with just TPD-FA; this attests to the potential for the use of nanophotosensitizers **34** as markers for imaging. The nanophotosensitizers designed here (**34**) display both two-photon excited therapeutic and imaging potential, with enhancement due to the improved FRET between the PPBF to the TPP and TPD moieties; those without the conjugated polymer are ca. 149 times less efficient. These nanophotosensitizers have potential to be used for concurrent two-photon PDT and fluorescence imaging, displaying both target specificity of cancer cells and low dark toxicity.

**Figure 16:**
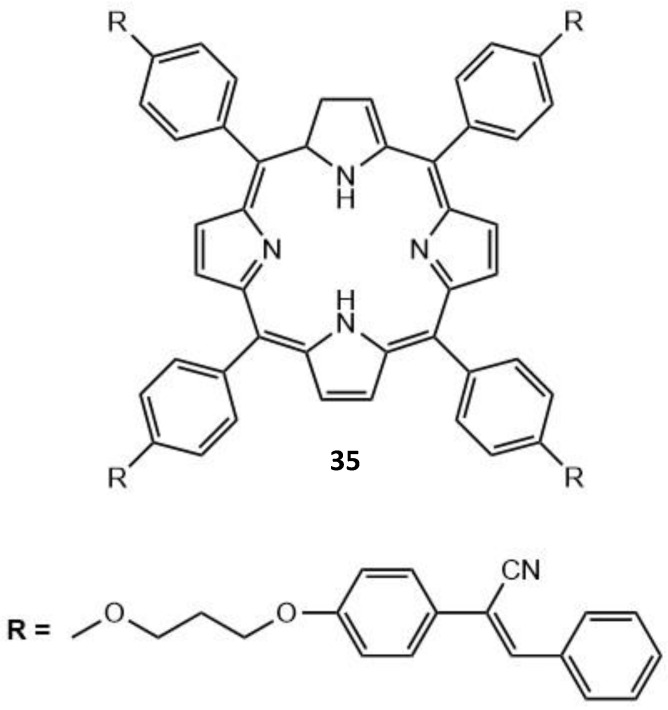
Structure of the novel AIE-based porphyrin (**35**).

A porphyrin derivative with aggregation-induced emission (AIE) properties has been developed (**35**, Figure 16); this was done via the introduction of diphenyl acrylonitrile units onto a porphyrin core [162]. A strong FRET effect was shown between the diphenyl acrylonitrile and porphyrin units; this led to the AIE-based porphyrin displaying high fluorescence in both the solution and solid states established due to the AIE and FRET effect.

This novel AIE-based porphyrin was also successfully utilized within living HeLa cells for fluorescence imaging, demonstrating a prospective application for this type of molecule. These fluorescence imaging properties were also shown to be an improvement on traditionally used aggregation-caused quenching porphyrins. The AIE-based porphyrin displayed a long emission wavelength, with both high photostability and fluorescence imaging capabilities, and biocompatibility, demonstrating the excellent prospects for near-infrared fluorescence imaging of living cells. Further studies will need to be done to see if these types of aggregates could have any therapeutic application combined with their imaging capabilities. This shows the wide range of molecules that can be utilized within the AIE fluorophore scope and the wide range of fields to which these molecules can be applied.

### 4.5. Large PEGylated Photosensitizers

**Figure 17:**
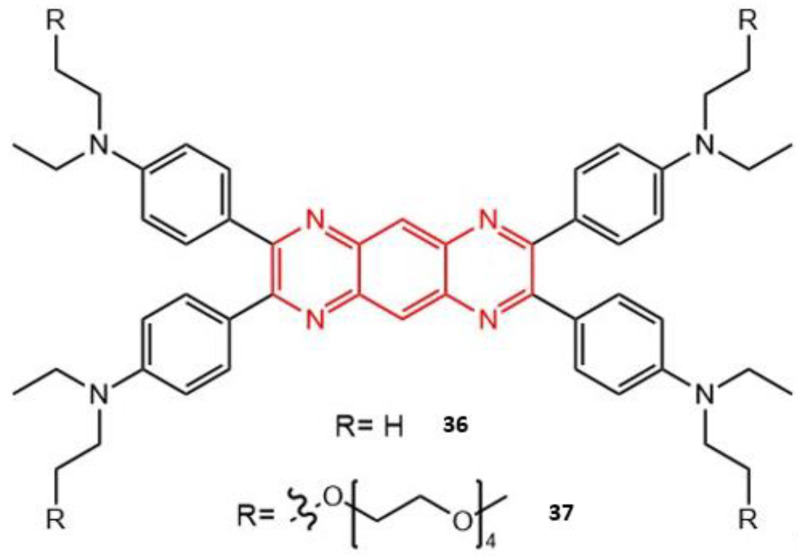
Structures of **36** and **37**.

Zhang et al. designed and synthesized a water-soluble photosensitizer (**37**) [163], the structure of which was based on a novel pyrazino [2,3-g] quinoxaline (PQ) prototype compound (**36**). PQ contains an aromatic skeleton featuring a tricyclic planar system in which a benzene ring is fused symmetrically with two pyrazine rings. Due to the electron-deficient and planar π-conjugated properties of PQ, it has been utilized as a building block for the construction of low band-gap small molecular- and polymeric-semiconductors used in the field of organic electroluminescence [164,165,166,167]. Then, the PQ moiety was modified with the addition of four pentaethylene glycol (PEG) groups (shown in red in Figure 17) to obtain the water-soluble **37**, allowing for good biocompatibility.

The PQ moiety acts as the central electron-deficient acceptor, with peripheral groups, N, N-diethyl phenylamine and PEG chain acting as the electron donors. The UV-Vis absorption and fluorescence emission of **37** were recorded in a mixture of THF and water, and with different ratios of water, the changes observed in both the absorption and emission showed the formation of aggregates with the increased ratio of water. The Փ_f_ of the two PSs, measured using Rhodamine B as the reference, amounted to 0.55 and 0.54 for **36** and **37**, respectively. The fluorescence lifetimes were also obtained, 1.99 ns for **36** in toluene and 2.0 ns and 1.5 ns, both for **37**, in toluene and water, respectively. The Փ_∆_ values were obtained by monitoring the decay of DPBF (in toluene) and found to be 0.49 and 0.47, for **36** and **37** respectively. The two-photon absorption spectra were measured within the 740–1000 nm range, using Rhodamine B as the standard reference [48]. Both PSs exhibited large cross-section values; the maxima were found at 820 nm with 1207 ± 69 GM for **36** and 1293 ± 72 GM for **37**. These TPA cross-section values, in combination with their respective Փ_f_ and Փ_∆_ values, are high enough for the simultaneous application of bioimaging and PDT. After irradiation with a high-intensity laser (532 or 635 nm, 100 mW/cm^2^, 30 mins), there was no significant photobleaching detected. One drawback was seen for **36**; due to the near planar central tricyclic PQ chromophore and the four peripheral N, N-diethyl phenylamines, this rigid and highly symmetric configuration favored the formation of π–π stacking aggregates. The substitution of four penta-PEG chains in the peripheral positions in **37** allowed more flexibility in the compound and not only provided adequate water solubility but also decreased the trend of π–π stacking. The results indicated that **37** was effectively taken up by tumor cells with excellent mitochondrial-targeting abilities [168,169]. The compound can effectively induce cell death under both one-photon excitation (1PE) and two-photon excitation (2PE). Good performance in cell imaging was observed under both one-photon (635 nm) and two-photon (800–1000 nm) excitation. Further tests revealed that the cytotoxicity of **37** was negligible in the dark, even with high concentrations of 100 μM. 

## 5. Nanoparticles as Two-Photon Absorption Species 

### 5.1. Polymer Nanoparticles

**Figure 18:**
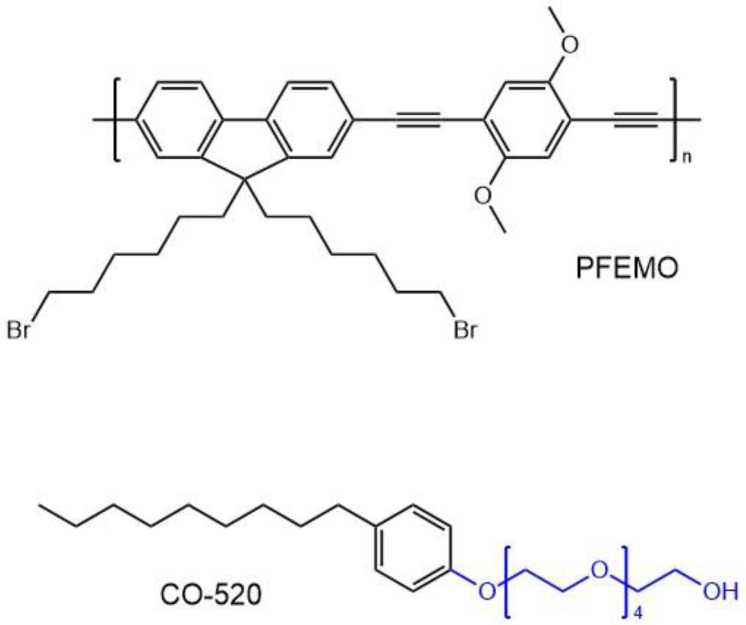
Structure of the components of PS-doped conjugated polymer nanoparticles (**38**).

The encapsulation of porphyrins and other tetrapyrrolic-based PSs in nanoparticles (NPs) is a good way to overcome certain drawbacks of large, organic molecules. In this context, the use of conjugated polymers is of special interest. With excellent biocompatibility, high fluorescence quantum yields, and large extinction coefficients [170,171,172], conjugated polymers have gained a lot of attention for their potential in clinical applications [173]. Several studies were concerned with utilizing the combination of such polymers and PSs, such as in the report by Shen et al [174]. The authors studied nanoparticles resulting from the combination of poly[9,9-dibromohexylfluorene-2,7-ylenethylene-alt-1,4-(2,5-dimethoxy) phenylene] (PFEMO) as the polymer moiety, and the PS used was the well-known TPP. An extra donor moiety, polyoxyethylene nonylphenylether (CO-520), was conjugated to TPP in order to enhance the TPA properties of the porphyrin by providing extra conjugation. The structure of these three components can be seen in Figure 18.

The two-photon excited emission of the nanoparticles, originating from TPP, was found to be enhanced by a factor of ca. 20 upon the addition of the PFEMO polymer. Recently, water-dispersible conjugated polymer nanoparticles were successfully prepared via mini-emulsion and re-precipitation methods [175,176,177]. Conjugated polymer nanoparticles were also constructed by blending two polymers or doping with specific dyes that can be tuned to alter their excitation and emission properties [178,179,180,181]. The PFEMO conjugated polymer can act as both the hosting material and the two-photon light-harvesting complex. The TPA cross-section of the doped conjugated polymer nanoparticles was measured to be 2160 GM per molecule (and 54 GM per repeating unit) at 800 nm. TPP was chosen as the photosensitizer moiety because of the good overlap of its Soret and Q-band absorption with the emission spectrum of PFEMO. The average size of the nanoparticles (**38**) was determined to be 80 nm by dynamic light scattering. Nanoparticles without PFEMO displayed only a weak emission peak at 652 nm due to the small TPA cross-section of TPP, this being in contrast to the emission peak of **38** containing the same concentration of TPP. The singlet oxygen generation of **38** was monitored by measuring its phosphorescence at 1270 nm with D_2_O being used as the solvent, which extends the emission lifetime of ^1^O_2_ to ca. 67 μs, in comparison to ca. 3.5 μs in H_2_O, as well as increases its emission yield [182]. The observed two-fold enhancement of singlet oxygen emission in **38**, compared to the nanoparticles without PFEMO, further confirms that PFEMO could act as an efficient light-harvesting component to enhance the photosensitizing capability of TPP via energy transfer. This significant enhancement of the two-photon singlet oxygen generation is also due to the large TPA cross-section of PFEMO. The nanoparticles were shown to have low cytotoxicity in the dark, ensuring their biological application potential. The incorporation of the CO-520 chains onto the surface of the nanoparticles ensures good stability, particularly for cellular applications. While this work has demonstrated two-photon photodynamic therapy on living cancer cells using photosensitizer-doped conjugated polymer nanoparticles as novel photosensitizing agents, there can be further optimizations to improve the cellular uptake efficiency, biocompatibility, and cancer cell targeting capability via the introduction of various functional groups onto the surface of the nanoparticles. Due to the strong two-photon excited emission of the used conjugated polymers, simultaneous two-photon imaging of the cells and monitoring of the distribution of the photosensitizing drug molecules was possible.

**Figure 19:**
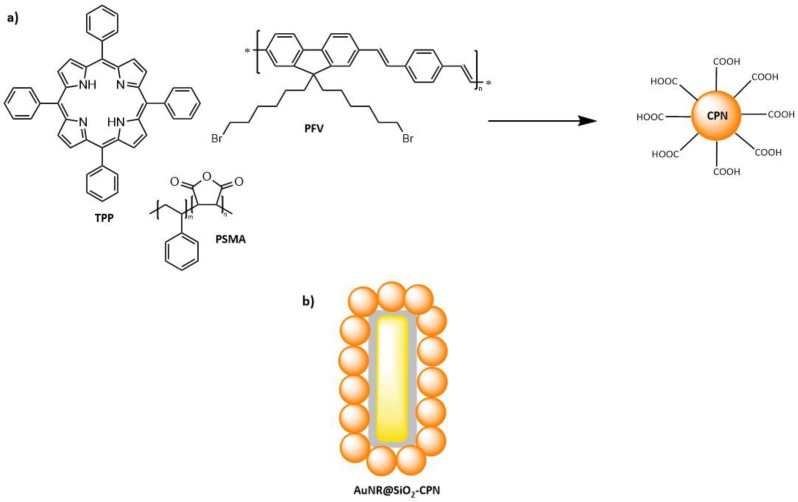
(**a**) Composition of the CPNs (**39**) and (**b**) Scheme of AuNR@SiO_2_-CPNs (**39**).

In a next example, several approaches to formulating a better two-photon PS have been taken together: a conjugated polymer, a photosensitizer, and gold nanorods (AuNRs) were combined in order to be used for TPA-PDT application (Figure 19) [183]. The well-studied photosensitizer, TPP, was encapsulated in poly[9,9’-bis(6”-bromohexyl)fluorene-2,7-ylenevinylene-co-alt-1,4-phenylene] (PFV), a conjugated polymer, with PMSA used as surfactant, to form conjugated polymer nanoparticles (CPNs); these CPNs (**39**) were covalently linked to AuNRs, which have been coated in silica (thickness of 9 nm). The improvement of two-photon absorption cross-sections in metal-containing nanoparticles has been well recognized [184,185,186].

The encapsulation of photosensitizers into nanoparticles is one way to overcome problems with the aggregation of PSs that takes place in aqueous media [187,188]. TPP is a well-known hydrophobic PS with a high Φ_Δ_ (singlet oxygen quantum yield) = 0.67, but its two-photon absorption cross-section is quite small, 8–12 GM [189,190]. PFV, a conjugated polymer, apart from being the host for TPP, can also have a role of a light-harvesting component through two-photon absorption. This polymer has a relatively large two-photon cross-section of 256 GM (at 820 nm) per each repeating unit and is capable of fluorescence energy transfer to TPP. The conjugated polymer-based NPs have been shown to exhibit excellent photophysical properties such as high brightness, good photostability, biocompatibility, easily tuned photophysical properties, and surface modification [170,191,192,193]. Thus, upon encapsulation, the two-photon optical properties of the system are enhanced. The third component of the system is the gold nanorods that display chemical inertness and biocompatibility, and they also have tunable extinction properties as well as strong two-photon excited photoluminescence [194]. AuNRs alone have been known to be able to produce ^1^O_2_, but as previously mentioned, they can also lead to the enhancement of ^1^O_2_ production by the PS in their vicinity due to their localized surface plasmon resonance (LSPR) properties (discussed in more detail in the following section) [195,196,197,198].

### 5.2. Gold Nanoparticles

Elemental gold, in the form of bulk solid or nanostructures, is well-known to be biocompatible and chemically inert. Gold nanostructures of various kinds, nanoparticles or nanostructured surfaces, can support conduction electrons’ excitations known as localized surface plasmons, which have potential applications, in e.g., bioimaging [199], as biosensors [200], for drug delivery [183], in photovoltaics [201], and also in PDT. Gold nanoparticles (AuNPs) are also known for their ability to be highly functionalized as well as their low cytotoxicity [202,203].

LSPR associated with the metal nanostructure can lead to the enhancement of fluorescence of weakly emitting fluorophores in its close proximity [204]. This enhancement can occur due to two factors: (1) increased absorption equating to excitation enhancement and (2) modified radiative and non-radiative decay rates equating to fluorescence emission enhancement [205]. The first effect is maximized when the plasmon resonance wavelength overlaps with the absorption band of the fluorophore/PS. The second effect is at maximum enhancement when the resonance wavelength overlaps with the emission band of the fluorophore/PS [206]. These enhancement factors can be dependent on several parameters such as the size and shape of the NP, composition of the NP, position of the fluorophore/PS in relation to the metal NP, and the spectral overlap of the relevant absorption and emission spectra [205,206,207,208]. A maximum enhancement was found to be when the plasmonic resonance of the metal nanoparticle matched the laser source operating wavelength [209]. However, there is also another effect that acts in the opposite way, namely, fluorescence quenching by the metal NPs, which occurs when the fluorophore/photosensitizer is too close to the metal NP, typically less than 5 nm [210].

**Figure 20:**
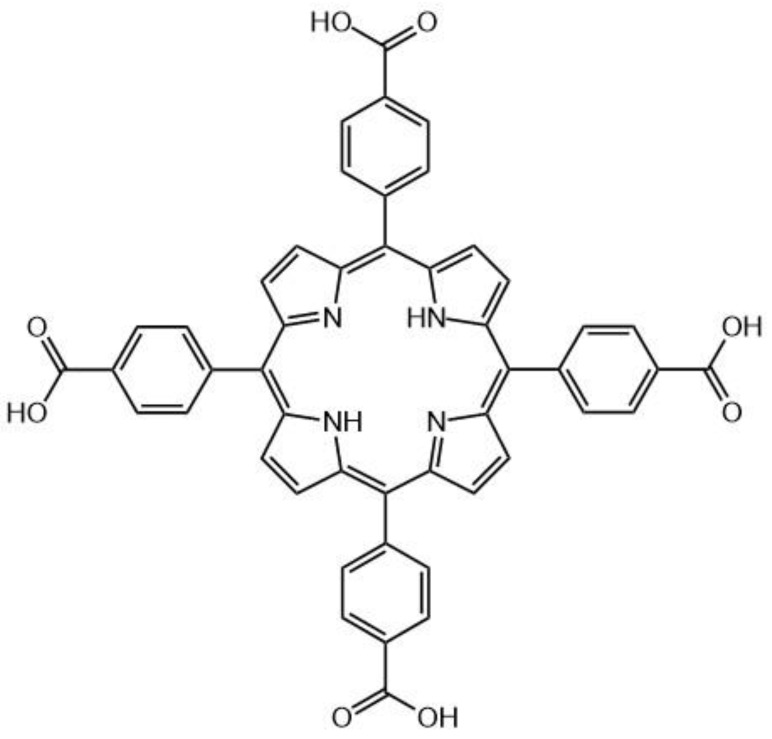
Structure of T790 (meso-tetra(4-carboxyphenyl) porphyrin).

In general, gold (or other metal) nanoparticles have been demonstrated to have positive effects on the optical properties of a PS through electric field amplification as well as the enhancement of the radiative and non-radiative decay rates [211]. In previous studies, it has been found that AuNRs are able to modulate the TPEF of neighboring fluorophores via modulation of the quantum yield combined with the enhancement of the two-photon excitation efficiency [212,213]. This has been tested by using several thicknesses of silica shell deposited on an NR, acting as a spacer between the metal surface and the fluorophore [214]. It was found that a thinner (<10 nm) SiO_2_ shell was more desirable for more optimal TPEF but proved more difficult to prepare. The increased amount of surfactant (cetyltrimethylammonium bromide), on the surface of the AuNRs, was shown to produce thinner silica shells. A study by Zhao et al. showed that it was possible to prepare AuNRs having a silica coating of between 13 and 42 nm thickness separating the Au core and the fluorophore, T790 (meso-tetra(4-carboxyphenyl) porphyrin, Figure 20) [215]. A thickness of 20 nm demonstrated the greatest enhancement of ^1^O_2_ generation efficiency via two-photon excitation with a simultaneous enhancement in TPEF.

#### 5.2.1. Gold Nanorods 

**Figure 21:**
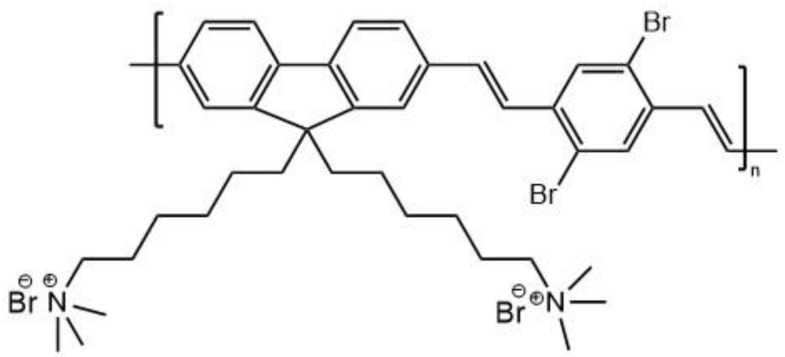
Structure of oligomer **40**.

In another study of gold nanorods combined with conjugated oligomers, it was also hoped that such combination may reduce the photodegradation of the PS molecules whilst preserving their photosensitizing capabilities as well as improving their solubility in biological media [201]. A core–shell nanostructure with an AuNR core and a silica shell whose thickness was controllable and an outer layer of a water-soluble oligomer, oligo-[9,9-bis(600-(N,N,N-trimethylammonium)hexyl)fluorene-2,7-ylenevinylene-co-alt-1,4-(2,5-dibromophenylene)] (**40**) (Figure 21), was investigated.

The oligomer was designed to have a large two-photon absorption cross-section and high singlet oxygen quantum yield. The fluorescence quantum yield of **40** was calculated to be 0.115 in water and 0.48 in methanol. The two-photon absorption cross-section was measured to be ≈280 GM (at 800 nm) per unit (mer). This relatively low value has been ascribed to the presence of the bromine, as this distorts the planarity of the molecule. The singlet oxygen quantum yield of **40** was measured to be 0.46. With the shell thickness at 15 nm (SiO_2_) in the AuNR/SiO_2_ core–shell, an enhancement factor of 14.2 in TPEF of **40** was determined. Since the noble metal nanoparticles can influence the fluorescence intensity of the PS-type molecule in their vicinity through three mechanisms [214,216,217], the question was which ones were responsible for this large enhancement. The amplification of the local electric field due to the plasmon resonance, which improves the excitation efficiency, largely depends on the overlap between the plasmon resonance and the excitation wavelength; however, this effect should have no effect on the lifetime of fluorescence of the PS. The plasmon resonance also increases the radiative decay rate of the nearby PS-type molecules, which enhances their fluorescence quantum yields but will also reduce the fluorescence lifetimes. This effect relies on the spectral overlap between the plasmon resonance band of the metal nanorods and the emission spectrum of the PS. A third effect is the energy transfer from the PS to the metal nanoparticle, which usually results in the quenching of fluorescence and reduced fluorescence lifetimes of the PS. All of these effects are dependent on the distance between the PS and metal nanoparticle. The optimum distance between the two components was found to be 15 nm. Therefore, such a core–shell structure was made, and **40** was adsorbed onto the surface of the silica-coated AuNRs via electrostatic attraction. The already mentioned enhancement of the TPEF by a factor of 14.2 was found to be due to the amplification of the electric field. 

#### 5.2.2. Gold Nanoclusters

**Figure 22:**
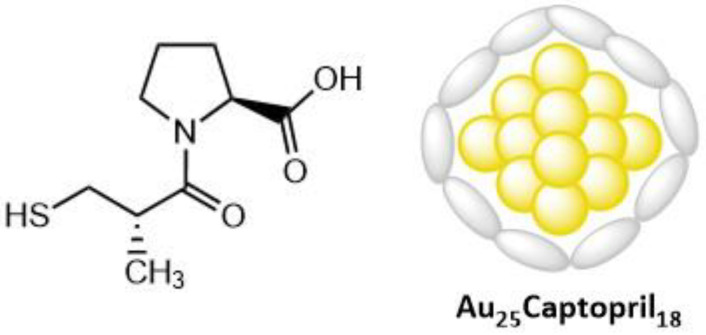
Structure of Captopril (**left**) and scheme of the Au_25_(Captopril)_18_ nanoclusters **41** (**right**).

Recent studies have shown the promise of gold nanoclusters (AuNCs) as a PS component of PDT; they are composed of several tens of gold atoms, have an average diameter of <2 nm [218], and are too small to support plasmon excitations. Gold (Au_25_(Captopril)_18_) nanoclusters (**41**) (Figure 22) are a new generation of water-soluble atomically precise gold nanoclusters, having improved thermal stability. They also demonstrate the generation of ROS under two-photon excitation; however, they were found to have low cellular uptake, limiting their potential for biomedical application. However, an improvement of the biocompatibility of these nanoclusters was reported upon incorporating them in hydrogel nanoparticles [219]. Polyacrylamide nanohydrogels (PAAm) have been previously shown to be useful at improving the biocompatibility of photodynamic agents by encapsulation [220]. These nanohydrogels have demonstrated the capacity to maintain the photodynamic properties of PS-type compounds (methylene blue), due to their highly stable solvent environment [221].

**Figure 23:**
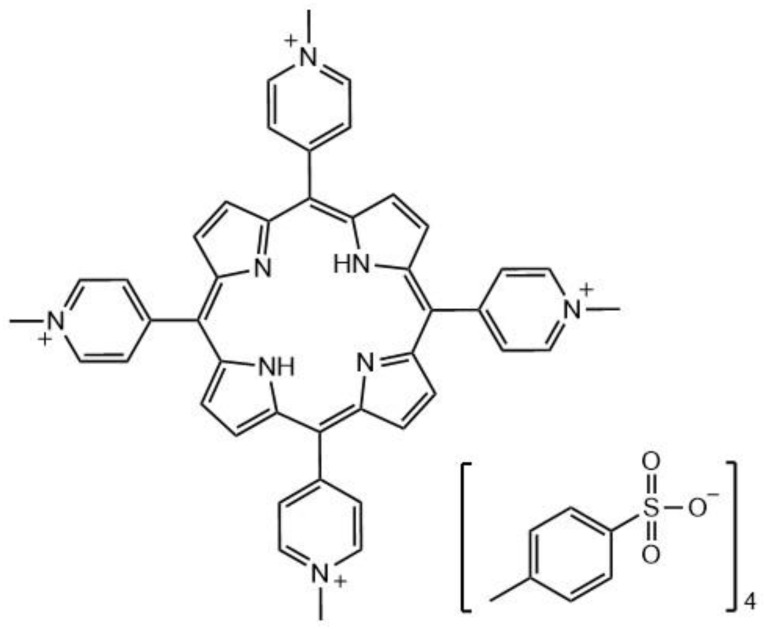
Structure of TMPyP.

It was found that the encapsulated nanoclusters had greater in vitro cellular uptake and were more easily conjugated to cell surfaces, contributing to the overall improvement of the biocompatibility. Two-photon absorption and the singlet oxygen generation capabilities of the nanoclusters were preserved when encapsulated. The two-photon absorption cross-section of **41** (Au_25_(Captopril)_18_) was determined via Z-scan to be 830 GM (λ = 800 nm) [222]. The singlet oxygen yield was also determined to be 1.6% for **41** and 1.4% for PAAm-Au_25_(Captopril)_18_ (**42**) by comparing to 5,10,15,20-Tetrakis(1-methyl-4-pyridinio)porphyrin tetra(p-toluenesulfonate (TMPyP, Figure 23), a known PS for which it is 18.2%. Although the singlet oxygen efficiencies are much lower than that for the reference, the two-photon absorption cross-section value is ≈8x higher than that of TMPyP. The gold nanoclusters also show potential as X-ray contrast agents (there are many examples in the literature that demonstrate how gold-containing compounds enhance the scattering of X-rays [223,224]); thus, they display a theranostic property that TMPyP and many other organic PSs do not possess. The non-encapsulated clusters **41** (without PAAm) were tested with HeLa 229 cancer cells in the dark for 24 hours; the cells displayed approximately 50% viability at concentrations as low as 0.1 mg/mL. However, when the encapsulated nanoclusters **42** were tested, the cells demonstrated >85% viability at concentrations of up to 10 mg/mL. The cells that were incubated with nanoparticles **42** showed no cell death with one-photon excitation, even after 30 minutes of irradiation. Upon two-photon irradiation of **42**, there was a much higher rate of cell death; >99% of the cells in the irradiated region were dead after 30 minutes (800 nm, 100 mW/cm^2^ (average power)). Due to the properties of gold nanoclusters, these nanoparticles could also potentially be used as CT contrast agents for soft tumors. 

#### 5.2.3. Gold Nanobipyramids

**Figure 24:**
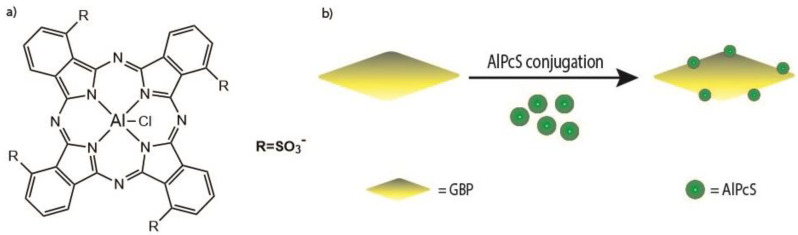
(**a**) Structure of sulfonated Al-phthalocyanine (AlPcS). (**b**) Schematic of the conjugation between the gold nanobipyramids and AlPcS (**43**).

An example of the use of gold nanostructures in two-photon PDT is a study where sulfonated aluminum phthalocyanine (AlPcS **43** conjugated to gold nanobipyramids (GBPs)), the structure shown in Figure 24, was excited via a two-photon process [225]. AlPcS has two absorption bands, at ca. 355 nm and 670 nm, with the fluorescence band at ca. 680 nm. The sulfonated phthalocyanine core has four negative charges, which can easily bind to the surface of the positively charged gold nanobipyramids to form conjugates. The AlPcS-GBP conjugates showed a large two-photon absorption cross-section, 26,000 ± 4000 GM (compared to 1200 ± 300 GM for gold nanospheres and 3700 ± 1100 GM for gold nanorods, all measured in aqueous solution, 800 nm) as well as good photostability and biocompatibility. Al-phthalocyanine alone has a measured TPA cross-section of 543 ± 16 GM (800 nm, in ethanol) [226]). ^1^O_2_ generation by **43** was confirmed by monitoring the decomposition of DPBF (λ_em_ = 410 nm). It was also confirmed by the ESR technique using TEMP as a ^1^O_2_ trap. 5,5-Dimethyl-1-pyrroline-N-oxide was utilized to confirm that no other ROS species were being generated (O_2_^●−^ or ^●^OH); no signal was observed, so it was confirmed that only ^1^O_2_ was being generated under two-photon excitation (800 nm). Under 800 nm fs laser excitation, efficient antitumor activity was observed both in vivo and in vitro.

### 5.3. Hybrid Nanoparticles

Hybrid CdS-Au nanoparticles (NPs) were studied by Nawrot et al. (**44**) [227]. Two-photon excited luminescence measurements were performed on the **44** NPs, and their TPA cross-section was determined. The obtained water-soluble NPs **44** were found to be multifunctional: as photocatalysts for both reduction and oxidation reactions, and for TPA applications, including theranostic purposes. ROS generation by **44** was observed, which is relevant for the use of such metal/semiconductor nanoparticles as possible antimicrobial agents for tumor cell death in targeted therapies.

The TPA cross-section value for **44** was determined to be 15.8 × 10^3^ GM at 725 nm, which exceeded the value observed for the non-metalated CdS NPs. This increase was attributed to the overlap of the semiconductor exciton band edge absorption and the plasmon resonance. Embedding of the gold nanostructures on the CdS NPs not only significantly enhanced their reduction and oxidation capabilities but also preserved their two-photon brightness, allowing the material to perform multiple functions.

### 5.4. Nanocontainers with Nanoparticles and Photosensitizers

**Figure 25:**
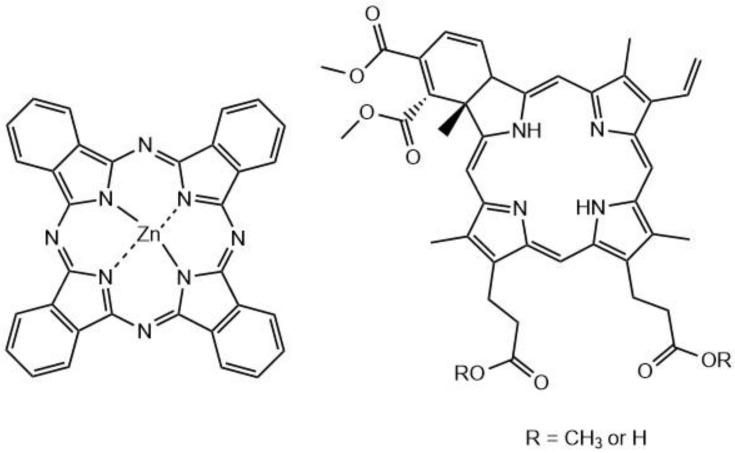
Structure of zinc (II) phthalocyanine (**left**) and verteporfin (**right**).

Another approach of using FRET, different from that described in Section 4.4, was demonstrated in several papers from the Wilk group [228,229,230]. The principle employed here was that of splitting the functions of a TPA absorber and that of a ROS generator. To assure that a two-photon absorber will transfer its excitation energy to a photosensitizer (e.g., phthalocyanine or verteporfin, Figure 25), both components of the FRET system were encapsulated inside relatively large (≈100 nm) nanocapsules. Such a size appears suitable for efficient PDT because of the so-called EPR (enhanced permeability and retention) effect [231,232], and it provides a possibility of having a large number, e.g., on the order of hundreds, nanoparticles embedded in a single nanocarrier. This results in entities that have cross-sections that are orders of magnitude larger than a single nanoparticle, thus, being very bright markers for two-photon-based imaging. The two-photon absorbers utilized in that approach were nanoparticles, which could be core–shell quantum dots that have relatively large two-photon absorption cross-sections in the 800–1000 nm region, or, importantly, lanthanide-based nanoparticles in which photon upconversion occurs [233,234,235,236,237]. In the latter case, the process that is in the end responsible for the excitation of the photosensitizer is not a direct two-photon absorption but rather a sequential process involving the lanthanide ions. This has an important advantage of not requiring femtosecond laser excitation but just pumping the lanthanide (ytterbium in that case) at its absorption band in the NIR with a continuous wave (CW) laser diode [238,239,240].

## 6. Summary and Outlook

In recent years, many developments of TPA capabilities of various molecules for PDT, bioimaging, optoelectronics, drug delivery, and more, have been made. This review provided certain examples of recent strategies used to improve the TPA properties, and two-photon induced ^1^O_2_ or other ROS types generation, of various chromophores and molecules. Table 1 provides a summary of the relevant properties of the photosensitizers described in this review. The relationship between the structure and the photophysical properties of these compounds has been described. To improve the TPA properties, the compounds presented here have increased π-conjugated systems for improved TPA cross-section values, improvements in SOC and ISC pathways, as well as being more highly branched for improved planarity and solubility (namely in aqueous media). The advantages of combining TPA with PDT can only be exploited with photosensitizers having large TPA cross-sections within the optimal biological window (700–1100 nm) and high ROS quantum yields. TPA-PDT prospects have been greatly improved by the use of highly conjugated and highly branched molecules, although the introduction of heteroatoms has also displayed unique possibilities for the use of the well-designed photosensitizers within TPA therapy and diagnostics. Metalated and non-metalated chromophores present additional choices for PSs for PDT/fluorescence imaging. Other modifications, such as photosensitizers/chromophores combined with various gold-based nanostructures, have also improved the prospects of TPA-PDT and imaging, due to the LSPR effect. Furthermore, there is also the interesting prospect of AIEgens for imaging-guided PDT. Improvements in the designs of these molecules have enhanced their ROS generation abilities with NIR excitation, thus improving the overall TPA-PDT prospect. 

The combination of TPA and PDT is invaluable for its reduced photothermal damages during treatment, along with its safer higher thresholds due to the femtosecond NIR laser source and its overall improved singlet oxygen quantum yields. Ensuring large TPA cross-sections with high ^1^O_2_ quantum yields and thus more efficient PDT has opened the doors for the combined use of TPA-PDT applications. The successful modifications of these TPA chromophores combined with the ever-advancing technology of medical devices and techniques could lead to the treatment of a wider range of cancers, particularly deeper-lying tumors, in a non-invasive manner. Regarding the future outlook of the types of TPA molecules, much progress has been made on their structure–photophysical property relationship; however, the following points require further investigation. (1) First, the increased use of DFT and TD-DFT calculations should provide information not only on the singlet and triplet energies (as well as ISC efficiency) but also how easily the molecules can be tuned. (2) Second, there ought to be more biological studies, as more information is required about how PS molecules behave in vivo and whether their efficiency and high-quantum yields in organic media translate to TPA-PDT application.

## Figures and Tables

**Table 1:** Summary of photophysical parameters of the molecules described in this review.

Molecule Number	λ_ex_ (nm)	σ_2_ (GM)	Φ_Δ_	Φ_F_	Page Number
**1**	700–1000	400	-	-	7
**2**	700–1000	1000	-	-	7
**3**	800	200	-	-	8
**4**	800	31	-	-	8
**5**	710	270	0.12	0.12	10
**6**	710	2800	0.89	0.93	10
**7**	-	-	-	-	10
**8**	720	4857.4	-	-	10
**9**	760	522	-	2.0 × 10^−4^	11
**10**	760	492	-	6.0 × 10^−5^	11
**11**	800	245	0.75	-	12
**12**	705	10.58	-	-	12
**13**	705	98.9	-	-	12
**14**	910	4000	0.38	-	13
**15**	910	21,500	0.19	-	13
**16**	725	3115	0.47	-	15
**17**	775	3700	0.27	-	15
**18**	800	730	0.73	0.04	15
**19**	800	580	0.50	0.025	15
**20**	820	1000	-	0.59	17
**21**	820	880	-	0.62	17
**22**	820	1100	-	0.55	17
**23**	820	750	-	0.63	17
**24**	820	1600	-	0.48	17
**25**	820	1750	-	0.52	17
**26**	820	1100	-	0.36	17
**27**	820	980	-	0.38	17
**28**	808	25.5	0.39	-	17
**29**	-	-	0.31	-	17
**30**	-	-	0.33	-	17
**31**	740	112	0.57	-	19
**32**	740	95	0.66	-	19
**33**	-	-	-	-	20
**34**	750	8.6 × 10^6^	-	-	20
**35**	-	-	-	-	22
**36**	820	1207	0.49	0.55	23
**37**	820	1293	0.47	0.54	23
**38**	800	2160	-	-	24
**39**	820	256	-	-	25
**40**	800	280	0.46	0.48	27
**41**	800	830	0.16	-	28
**42**	-	-	0.14	-	28
**43**	800	26,000	-	-	30
**44**	725	15.8 × 10^3^	-	-	30

## Data Availability

Not applicable.

## Sample Availability

Samples of the compounds are not available from the authors.

## References

[1] Rumi M., Perry J.W. (2010). Two-photon absorption: An overview of measurements and principles. Adv. Opt. Photonics.

[2] Kobayashi Y., Mutoh K., Abe J. (2018). Stepwise two-photon absorption processes utilizing photochromic reactions. J. Photochem. Photobiol. C Photochem. Rev..

[3] Pawlicki M., Collins H.A., Denning R.G., Anderson H.L. (2009). Two-photon absorption and the design of two-photon dyes. Angew. Chem. Int. Ed. Engl..

[4] Kaiser W., Garrett C.G.B. (1961). Two-Photon Excitation in CaF_2_:Eu^2+^. Phys. Rev. Lett..

[5] Shen Y., Shuhendler A.J., Ye D., Xu J.J., Chen H.Y. (2016). Two-photon excitation nanoparticles for photodynamic therapy. Chem. Soc. Rev..

[6] McKenzie L.K., Bryant H.E., Weinstein J.A. (2019). Transition metal complexes as photosensitisers in one-and two-photon photodynamic therapy. Coord. Chem. Rev..

[7] Gu B., Wu W., Xu G., Feng G., Yin F., Chong P.H.J., Qu J., Yong K.T., Liu B. (2017). Precise two-photon photodynamic therapy using an efficient photosensitizer with aggregation-induced emission characteristics. Adv. Mater..

[8] Cumpston B.H., Ananthavel S.P., Barlow S., Dyer D.L., Ehrlich J.E., Erskine L.L., Heikal A.A., Kuebler S.M., Lee I.Y.S., McCord-Maughon D. (1999). Two-photon polymerization initiators for three-dimensional optical data storage and microfabrication. Nature.

[9] Zhang Q., Yue S., Sun H., Wang X., Hao X., An S. (2017). Nondestructive up-conversion readout in Er/Yb co-doped Na0.5Bi2.5Nb2O9-based optical storage materials for optical data storage device applications. J. Mater. Chem. C.

[10] Liu J.-C., Li X.-Z., Zhang Y. (2016). Two-photon absorption induced optical power limiting behavior of strong femtosecond hyper-Gaussian pulses. Proceedings of the SPIE/COS Photonics Asia.

[11] Chang Z.F., Jing L.M., Chen B., Zhang M., Cai X., Liu J.J., Ye Y.C., Lou X., Zhao Z., Liu B. (2016). Rational design of asymmetric red fluorescent probes for live cell imaging with high AIE effects and large two-photon absorption cross sections using tunable terminal groups. Chem. Sci..

[12] Zhang Q., Tian X., Zhou H., Wu J., Tian Y. (2017). Lighting the Way to See Inside Two-Photon Absorption Materials: Structure-Property Relationship and Biological Imaging. Materials.

[13] Lee J., Arita Y., Matsuo R., Kawaguchi H., Miyamoto K., Dholakia K., Omatsu T. (2019). Photopolymerization with Light Fields Possessing Orbital Angular Momentum: Generation of Helical Microfibers. Proceedings of the Lasers and Electro-Optics Europe & European Quantum Electronics Conference (CLEO/Europe-EQEC).

[14] Xu X., Madrigal J.B., Broussier A., Lio G.E., Geoffray F., Issa A., Jradi S., Bachelot R., Couteau C., Blaize S. (2020). Quantum emitters based on polymeric structures embedded with quantum dots fabricated via photo-polymerization. Advanced Fabrication Technologies for Micro/Nano Optics and Photonics XIII, Proceeding of SPIE OPTO, San Francisco, CA, USA, 28 February 2020.

[15] Niesler F., Hermatschweiler M. (2016). Two-Photon Polymerization - A Versatile Microfabrication Tool. Opt. Photonik..

[16] Lemma E.D., Spagnolo B., De Vittorio M., Pisanello F. (2019). Studying Cell Mechanobiology in 3D: The Two-Photon Lithography Approach. Trends Biotechnol..

[17] Scarpa E., Lemma E.D., Fiammengo R., Cipolla M.P., Pisanello F., Rizzi F., De Vittorio M. (2019). Microfabrication of pH-responsive 3D hydrogel structures via two-photon polymerization of high-molecular-weight poly(ethylene glycol) diacrylates. Sens. Actuators B Chem..

[18] Kikuchi K. (1998). Highly sensitive interferometric autocorrelator using Si avalanche photodiode as two-photon absorber. Electron. Lett..

[19] Hayakawa R., Ishikura N., Nguyen H.C., Baba T. (2013). Two-photon-absorption photodiodes in Si photonic-crystal slow-light waveguides. Appl. Phys. Lett..

[20] Homann C., Krebs N., Riedle E. (2011). Convenient pulse length measurement of sub-20-fs pulses down to the deep UV via two-photon absorption in bulk material. Appl. Phys. B.

[21] Fan W., Huang P., Chen X. (2016). Overcoming the Achilles’ heel of photodynamic therapy. Chem. Soc. Rev..

[22] Mazur L.M., Roland T., Leroy-Lhez S., Sol V., Samoc M., Samuel I.D.W., Matczyszyn K. (2019). Efficient Singlet Oxygen Photogeneration by Zinc Porphyrin Dimers upon One- and Two-Photon Excitation. J Phys. Chem. B.

[23] Frochot C., Mordon S. (2019). Update of the situation of clinical photodynamic therapy in Europe in the 2003–2018 period. J. Porphyr. Phthalocyanines.

[24] dos Santos A.F., de Almeida D.R.Q., Terra L.F., Baptista M.S., Labriola L. (2019). Photodynamic therapy in cancer treatment-an update review. J. Cancer Metastasis Treat..

[25] Li X., Lovell J.F., Yoon J., Chen X. (2020). Clinical development and potential of photothermal and photodynamic therapies for cancer. Nat. Rev. Clin. Oncol..

[26] Sun J., Kormakov S., Liu Y., Huang Y., Wu D., Yang Z. (2018). Recent progress in metal-based nanoparticles mediated photodynamic therapy. Molecules.

[27] Park Y.-K., Park C.-H. (2016). Clinical efficacy of photodynamic therapy. Obstet. Gynecol. Sci..

[28] Kwiatkowski S., Knap B., Przystupski D., Saczko J., Kędzierska E., Knap-Czop K., Kotlińska J., Michel O., Kotowski K., Kulbacka J. (2018). Photodynamic therapy–mechanisms, photosensitizers and combinations. Biomed. Pharmacother..

[29] Makarov N.S., Drobizhev M., Wicks G., Makarova E.A., Lukyanets E.A., Rebane A. (2013). Alternative selection rules for one-and two-photon transitions in tribenzotetraazachlorin: Quasi-centrosymmetrical π-conjugation pathway of formally non-centrosymmetrical molecule. J. Chem. Phys..

[30] Bhawalkar J., Kumar N., Zhao C.-F., Prasad P. (1997). Two-photon photodynamic therapy. J. Clin. Laser Med. Surg..

[31] Rocha L.B., Soares H.T., Mendes M.I.P., Cabrita A., Schaberle F.A., Arnaut L.G. (2020). Necrosis Depth and Photodynamic Threshold Dose with Redaporfin-PDT. Photochem. Photobiol..

[32] Paschotta R. (2008). Encyclopedia of Laser Physics and Technology, Vol. 1.

[33] Vivas M.G., De Boni L., Mendonça C.R., Gupta V.P. (2017). Molecular and Laser Spectroscopy: Advances and Applications.

[34] GeorgeáTruscott T., Edward J. (1990). Effect of oxygen-enhanced intersystem crossing on the observed efficiency of formation of singlet oxygen. J. Chem. Soc. Faraday Trans..

[35] McClure D.S. (1952). Spin-orbit interaction in aromatic molecules. J. Chem. Phys..

[36] Marian C.M. (2012). Spin–orbit coupling and intersystem crossing in molecules. Wiley Interdiscip. Rev. Comput. Mol. Sci..

[37] Koziar J.C., Cowan D.O. (1978). Photochemical heavy-atom effects. Acc. Chem. Res..

[38] Ohulchanskyy T.Y., Donnelly D.J., Detty M.R., Prasad P.N. (2004). Heteroatom substitution induced changes in excited-state photophysics and singlet oxygen generation in chalcogenoxanthylium dyes: Effect of sulfur and selenium substitutions. J. Phys. Chem. B.

[39] Wang C., Abbas M., Wantz G., Kawabata K., Takimiya K. (2020). “Heavy-atom effects” in the parent [1] benzochalcogenopheno [3, 2-b][1] benzochalcogenophene system. J. Mater. Chem. C.

[40] Martinez V., Henary M. (2016). Nile red and Nile blue: Applications and syntheses of structural analogues. Chem.-A Eur. J..

[41] Petri A., Yova D., Alexandratou E., Kyriazi M., Rallis M. (2012). Comparative characterization of the cellular uptake and photodynamic efficiency of Foscan® and Fospeg in a human prostate cancer cell line. Photodiagnosis Photodyn. Ther..

[42] da Silva C.L., Del Ciampo J.O., Rossetti F.C., Bentley M.V.L.B., Pierre M.B.R. (2013). PLGA nanoparticles as delivery systems for protoporphyrin IX in topical PDT: Cutaneous penetration of photosensitizer observed by fluorescence microscopy. J. Nanosci. Nanotechnol..

[43] Chen K. (2010). Photophysical Characterization and Optimization of Novel Polymer Based Photosensitizer Carrier Systems for PDT. Ph.D. Thesis.

[44] Yoon I., Li J.Z., Shim Y.K. (2013). Advance in photosensitizers and light delivery for photodynamic therapy. Clin. Endosc..

[45] Xu C., Webb W.W. (1996). Measurement of two-photon excitation cross sections of molecular fluorophores with data from 690 to 1050 nm. JOSA B.

[46] Mertz J., Xu C., Webb W. (1995). Single-molecule detection by two-photon-excited fluorescence. Opt. Lett..

[47] Drobizhev M., Tillo S., Makarov N., Hughes T., Rebane A. (2009). Absolute two-photon absorption spectra and two-photon brightness of orange and red fluorescent proteins. J. Phys. Chem. B.

[48] Makarov N.S., Drobizhev M., Rebane A. (2008). Two-photon absorption standards in the 550-1600 nm excitation wavelength range. Opt Express..

[49] Wang J., Sheik-Bahae M., Said A.A., Hagan D.J., Van Stryland E.W. (1994). Time-resolved Z-scan measurements of optical nonlinearities. J. Opt. Soc. Am. B.

[50] Sheik-Bahae M., Said A.A., Wei T.-H., Wu Y.-Y., Hagan D.J., Soileau M., Van Stryland E.W. (1990). Z-Scan: A Simple and Sensitive Technique for Nonlinear Refraction Measurements. Nonlinear Optical Properties of Materials, Proceeding of the 33rd Annual Technical Symposium, San Jose, CA, USA, 4 January 1990.

[51] DeSalvo R., Sheik-Bahae M., Said A., Hagan D.J., Van Stryland E.W. (1993). Z-scan measurements of the anisotropy of nonlinear refraction and absorption in crystals. Opt. Lett..

[52] Sheik-Bahae M., Wang J., DeSalvo R., Hagan D., Van Stryland E. (1992). Measurement of nondegenerate nonlinearities using a two-color Z scan. Opt. Lett..

[53] Dinesh Babu K., Murali K., Karthikeyan N., Karuppusamy S. (2019). Investigation of optical limiting and third-order optical nonlinear properties of 2-Nitroaniline by Z-scan and f-scan techniques. Laser Phys..

[54] Ajami A., Husinsky W., Tromayer M., Gruber P., Liska R., Ovsianikov A. (2017). Measurement of degenerate two-photon absorption spectra of a series of developed two-photon initiators using a dispersive white light continuum Z-scan. Appl. Phys. Lett..

[55] Gu B., Fan Y.-X., Chen J., Wang H.-T., He J., Ji W. (2007). Z-scan theory of two-photon absorption saturation and experimental evidence. J. Appl. Phys..

[56] Hu Y., Gu B., Wen B., Lv C., Rui G., He J., Cui Y. (2020). Anisotropic two-photon absorbers measured by the Z-scan technique and its application in laser beam shaping. J. Opt. Soc. Am. B.

[57] Garcia H., Serna J., Rueda E. (2020). Bulk ZnSe and CdS two-photon absorption measurement with an F-scan nonlinear absorption spectrometer. OSA Continuum..

[58] Kolkowski R., Samoc M. (2014). Modified Z-scan technique using focus-tunable lens. J. Optics..

[59] Rueda E., Serna J.H., Hamad A., Garcia H. (2019). Two-photon absorption coefficient determination using the differential F-scan technique. Opt. Laser Technol..

[60] Cronstrand P., Luo Y., Ågren H. (2005). Multi-photon absorption of molecules. Adv. Quantum Chem..

[61] Wang C.-K., Macak P., Luo Y., Ågren H. (2001). Effects of π centers and symmetry on two-photon absorption cross sections of organic chromophores. J. Chem. Phys..

[62] Salem M., Gedik M., Brown A., Leszczynski J., Kaczmarek-Kedziera A., Puzyn T., Papadopoulos M.G., Reis H., Shukla M.K. (2015). Two photon absorption in biological molecules. Handbook of Computational Chemistry.

[63] Nifosì R., Luo Y. (2007). Predictions of novel two-photon absorption bands in fluorescent proteins. J. Phys. Chem. B.

[64] Salem M.A., Brown A. (2014). Two-photon absorption in fluorescent protein chromophores: TDDFT and CC2 results. J. Chem. Theory Comput..

[65] Karotki A., Kruk M., Drobizhev M., Rebane A., Nickel E., Spangler C.W. (2001). Efficient singlet oxygen generation upon two-photon excitation of new porphyrin with enhanced nonlinear absorption. IEEE J. Sel. Top. Quantum Electron..

[66] Arnbjerg J., Johnsen M., Frederiksen P.K., Braslavsky S.E., Ogilby P.R. (2006). Two-photon photosensitized production of singlet oxygen: Optical and optoacoustic characterization of absolute two-photon absorption cross sections for standard sensitizers in different solvents. J. Phys. Chem. A..

[67] Poulsen T.D., Frederiksen P.K., Jørgensen M., Mikkelsen K.V., Ogilby P.R. (2001). Two-Photon Singlet Oxygen Sensitizers: Quantifying, Modeling, and Optimizing the Two-Photon Absorption Cross Section. J. Phys. Chem. A.

[68] Ishi-i T., Taguri Y., Kato S.-i., Shigeiwa M., Gorohmaru H., Maeda S., Mataka S. (2007). Singlet oxygen generation by two-photon excitation of porphyrin derivatives having two-photon-absorbing benzothiadiazole chromophores. J. Mater. Chem..

[69] Pitre S.P., McTiernan C.D., Vine W., DiPucchio R., Grenier M., Scaiano J.C. (2015). Visible-Light Actinometry and Intermittent Illumination as Convenient Tools to Study Ru(bpy)3Cl2 Mediated Photoredox Transformations. Sci. Rep..

[70] Darmanyan A.P. (1982). Generation of 1O2 and the mechanism of internal conversion in 9,10-diphenylanthracene. Chem. Phys. Lett..

[71] Schmitz C., Aubry J.M., Rigaudy J. (1982). A new access to the anthracene core. Tetrahedron.

[72] Fatima K., Masood N., Luqman S. (2016). Quenching of singlet oxygen by natural and synthetic antioxidants and assessment of electronic UV/Visible absorption spectra for alleviating or enhancing the efficacy of photodynamic therapy. Biomed. Res. Ther..

[73] Hartman P.E., Hartman Z., Ault K.T. (1990). Scavenging of singlet molecular oxygen by imidazole compounds: High and sustained activities of carboxy terminal histidine dipeptides and exceptional activity of imidazole-4-acetic acid. Photochem. Photobiol..

[74] Linetsky M., Ortwerth B.J. (1997). Quantitation of the singlet oxygen produced by UVA irradiation of human lens proteins. Photochem. Photobiol..

[75] Lion Y., Delmelle M., Van de Vorst A. (1976). New method of detecting singlet oxygen production. Nature.

[76] Abbas K., Babić N., Peyrot F. (2016). Use of spin traps to detect superoxide production in living cells by electron paramagnetic resonance (EPR) spectroscopy. Methods.

[77] Nakamura K., Ishiyama K., Ikai H., Kanno T., Sasaki K., Niwano Y., Kohno M. (2011). Reevaluation of analytical methods for photogenerated singlet oxygen. J. Clin. Biochem. Nutr..

[78] He W., Liu Y., Wamer W.G., Yin J.-J. (2014). Electron spin resonance spectroscopy for the study of nanomaterial-mediated generation of reactive oxygen species. J. Food Drug Anal..

[79] Fan J., Qin H., Jiang S. (2019). Mn-doped g-C3N4 composite to activate peroxymonosulfate for acetaminophen degradation: The role of superoxide anion and singlet oxygen. Chem. Eng. J..

[80] Igarashi T., Sakurai K., Oi T., Obara H., Ohya H., Kamada H. (1999). New sensitive agents for detecting singlet oxygen by electron spin resonance spectroscopy. Free Radic. Biol. Med..

[81] Jung M.Y., Choi D.S. (2010). Electron spin resonance and luminescence spectroscopic observation and kinetic study of chemical and physical singlet oxygen quenching by resveratrol in methanol. J. Agric. Food Chem..

[82] Kumar A., Prasad A., Pospíšil P. (2020). Formation of α-tocopherol hydroperoxide and α-tocopheroxyl radical: Relevance for photooxidative stress in Arabidopsis. Sci. Rep..

[83] Marchand G., Calliste C.A., Williams R.M., McLure C., Leroy-Lhez S., Villandier N. (2018). Acetylated lignins: A potential bio-sourced photosensitizer. ChemistrySelect.

[84] Ando T., Yoshikawa T., Tanigawa T., Kohno M., Yoshida N., Kondo M. (1997). Quantification of singlet oxygen from hematoporphyrin derivative by electron spin resonance. Life Sci..

[85] Matsumura Y., Iwasawa A., Kobayashi T., Kamachi T., Ozawa T., Kohno M. (2013). Detection of high-frequency ultrasound-induced singlet oxygen by the ESR spin-trapping method. Chem. Lett..

[86] Myung Kim H., Rae Cho B. (2009). Two-photon materials with large two-photon cross sections. Structure-property relationship. Chem. Commun..

[87] Terenziani F., Katan C., Badaeva E., Tretiak S., Blanchard-Desce M. (2008). Enhanced Two-Photon Absorption of Organic Chromophores: Theoretical and Experimental Assessments. Adv. Mater..

[88] Xu L., Zhang J., Yin L., Long X., Zhang W., Zhang Q. (2020). Recent progress in efficient organic two-photon dyes for fluorescence imaging and photodynamic therapy. J. Mater. Chem. C.

[89] Mongin O., Hugues V., Blanchard-Desce M., Merhi A., Drouet S., Yao D., Paul-Roth C. (2015). Fluorenyl porphyrins for combined two-photon excited fluorescence and photosensitization. Chem. Phys. Lett..

[90] Oar M.A., Serin J.M., Dichtel W.R., Fréchet J.M.J., Ohulchanskyy T.Y., Prasad P.N. (2005). Photosensitization of Singlet Oxygen via Two-Photon-Excited Fluorescence Resonance Energy Transfer in a Water-Soluble Dendrimer. Chem. Mater..

[91] Huang H., Yu B., Zhang P., Huang J., Chen Y., Gasser G., Ji L., Chao H. (2015). Highly Charged Ruthenium(II) Polypyridyl Complexes as Lysosome-Localized Photosensitizers for Two-Photon Photodynamic Therapy. Angew. Chem. Int. Ed. Engl..

[92] McKenzie L.K., Sazanovich I.V., Baggaley E., Bonneau M., Guerchais V., Williams J.A., Weinstein J.A., Bryant H.E. (2017). Metal Complexes for Two-Photon Photodynamic Therapy: A Cyclometallated Iridium Complex Induces Two-Photon Photosensitization of Cancer Cells under Near-IR Light. Chemistry.

[93] Dahlstedt E., Collins H.A., Balaz M., Kuimova M.K., Khurana M., Wilson B.C., Phillips D., Anderson H.L. (2009). One- and two-photon activated phototoxicity of conjugated porphyrin dimers with high two-photon absorption cross sections. Org. Biomol. Chem..

[94] Dy J.T., Ogawa K., Satake A., Ishizumi A., Kobuke Y. (2007). Water-soluble self-assembled butadiyne-bridged bisporphyrin: A potential two-photon-absorbing photosensitizer for photodynamic therapy. Chemistry.

[95] Reinhardt B.A., Brott L.L., Clarson S.J., Dillard A.G., Bhatt J.C., Kannan R., Yuan L., He G.S., Prasad P.N. (1998). Highly Active Two-Photon Dyes: Design, Synthesis, and Characterization toward Application. Chem. Mater..

[96] Albota M., Beljonne D., Bredas J.L., Ehrlich J.E., Fu J.Y., Heikal A.A., Hess S.E., Kogej T., Levin M.D., Marder S.R. (1998). Design of organic molecules with large two-photon absorption cross sections. Science.

[97] Ohta K., Kamada K. (2007). Theoretical Approach to Large Two-Photon Absorption Cross Section in Extended π-Conjugated Systems. AIP Conf. Proc..

[98] Karotki A., Kruk M., Rebane A., Nickel E., Charles W.S., Dougherty T.J. (2002). Strong two-photon absorption and singlet oxygen photogeneration in near-IR with new porphyrin molecule. Optical Methods for Tumor Treatment and Detection: Mechanisms and Techniques in Photodynamic Therapy XI, Proceedings of International Symposium on Biomedical Optics, San Jose, CA, USA, 6 June 2002.

[99] Wielgus M., Bartkowiak W., Samoc M. (2012). Two-photon solvatochromism. I. Solvent effects on two-photon absorption cross section of 4-dimethylamino-4′-nitrostilbene (DANS). Chem. Phys. Lett..

[100] Wielgus M., Zalesny R., Murugan N.A., Kongsted J., Agren H., Samoc M., Bartkowiak W. (2013). Two-photon solvatochromism II: Experimental and theoretical study of solvent effects on the two-photon absorption spectrum of Reichardt’s dye. Chemphyschem.

[101] Wielgus M., Michalska J., Samoc M., Bartkowiak W. (2015). Two-photon solvatochromism III: Experimental study of the solvent effects on two-photon absorption spectrum of p-nitroaniline. Dye. Pigment..

[102] Wielgus M., Samoć M., Bartkowiak W. (2016). Two-photon absorption of Crystal Violet in solutions: Analysis of the solvent effect and aggregation process based on linear and nonlinear absorption spectra. J. Mol. Liq..

[103] Hornum M., Reinholdt P., Zareba J.K., Jensen B.B., Wustner D., Samoc M., Nielsen P., Kongsted J. (2020). One- and two-photon solvatochromism of the fluorescent dye Nile Red and its CF3, F and Br-substituted analogues. Photochem. Photobiol. Sci..

[104] Sharmoukh W., Attanzio A., Busatto E., Etienne T., Carli S., Monari A., Assfeld X., Beley M., Caramori S., Gros P.C. (2015). 2,5-Dithienylpyrrole (DTP) as a donor component in DTP–π–A organic sensitizers: Photophysical and photovoltaic properties. RSC Adv..

[105] Sengul O., Marazzi M., Monari A., Catak S. (2018). Photophysical Properties of Novel Two-Photon Absorbing Dyes: Assessing Their Possible Use for Singlet Oxygen Generation. J. Phys. Chem. C.

[106] Sengul O., Boydas E.B., Pastore M., Sharmouk W., Gros P.C., Catak S., Monari A. (2017). Probing optical properties of thiophene derivatives for two-photon absorption. Theor. Chem. Acc..

[107] Turan H.T., Eken Y., Marazzi M., Pastore M., Aviyente V., Monari A. (2016). Assessing One- and Two-Photon Optical Properties of Boron Containing Arenes. J. Phys. Chem. C.

[108] Gallavardin T., Armagnat C., Maury O., Baldeck P.L., Lindgren M., Monnereau C., Andraud C. (2012). An improved singlet oxygen sensitizer with two-photon absorption and emission in the biological transparency window as a result of ground state symmetry-breaking. Chem. Commun..

[109] Hu W., Xie M., Zhao H., Tang Y., Yao S., He T., Ye C., Wang Q., Lu X., Huang W. (2018). Nitric oxide activatable photosensitizer accompanying extremely elevated two-photon absorption for efficient fluorescence imaging and photodynamic therapy. Chem. Sci..

[110] Hu W., He T., Jiang R., Yin J., Li L., Lu X., Zhao H., Zhang L., Huang L., Sun H. (2017). Inner salt-shaped small molecular photosensitizer with extremely enhanced two-photon absorption for mitochondrial-targeted photodynamic therapy. Chem. Commun..

[111] Zheng M.L., Fujita K., Chen W.Q., Smith N.I., Duan X.M., Kawata S. (2011). Comparison of staining selectivity for subcellular structures by carbazole-based cyanine probes in nonlinear optical microscopy. ChemBioChem.

[112] Zheng Y.-C., Zheng M.-L., Chen S., Zhao Z.-S., Duan X.-M. (2014). Biscarbazolylmethane-based cyanine: A two-photon excited fluorescent probe for DNA and selective cell imaging. J. Mater. Chem. B..

[113] Zheng Y.-C., Zheng M.-L., Li K., Chen S., Zhao Z.-S., Wang X.-S., Duan X.-M. (2015). Novel carbazole-based two-photon photosensitizer for efficient DNA photocleavage in anaerobic condition using near-infrared light. RSC Adv..

[114] Sajewicz W., Dlugosz A. (2000). Cytotoxicity of some potential DNA intercalators (carbazole, acridine and anthracene derivatives) evaluated through neutrophil chemiluminescence. J. Appl. Toxicol..

[115] Gu J., Yulan W., Chen W.-Q., Dong X.-Z., Duan X.-M., Kawata S. (2007). Carbazole-based 1D and 2D hemicyanines: Synthesis, two-photon absorption properties and application for two-photon photopolymerization 3D lithography. New J. Chem..

[116] Taima H., Okubo A., Yoshioka N., Inoue H. (2006). DNA-binding properties and photocleavage activity of cationic water-soluble chlorophyll derivatives. Chemistry.

[117] Chen L.L., Zheng M.L., Zheng Y.C., Jin F., Chai Q.Q., Zhao Y.Y., Meng X.W., Liu Y.H., Duan X.M. (2018). Laser-Induced Antibacterial Activity of Novel Symmetric Carbazole-Based Ethynylpyridine Photosensitizers. ACS Omega.

[118] Gluszynska A. (2015). Biological potential of carbazole derivatives. Eur. J. Med. Chem..

[119] Knolker H.J., Reddy K.R. (2002). Isolation and synthesis of biologically active carbazole alkaloids. Chem. Rev..

[120] Chen Y., Yamamura T., Igarashi K. (2000). Photosensitization of carbazole derivatives in cationic polymerization with a novel sensitivity to near-UV light. J. Polym. Sci. Part A Polym. Chem..

[121] Grigalevicius S. (2006). 3,6(2,7),9-Substituted carbazoles as electroactive amorphous materials for optoelectronics. Synth. Met..

[122] Wasielewski M.R., Svec W.A. (1980). Synthesis of covalently linked dimeric derivatives of chlorophyll a, pyrochlorophyll a, chlorophyll b, and bacteriochlorophyll a. J. Org. Chem..

[123] Lower S.K., El-Sayed M.A. (1966). The Triplet State and Molecular Electronic Processes in Organic Molecules. Chem. Rev..

[124] Susumu K., Fisher J.A., Zheng J., Beratan D.N., Yodh A.G., Therien M.J. (2011). Two-photon absorption properties of proquinoidal D-A-D and A-D-A quadrupolar chromophores. J. Phys. Chem. A.

[125] Schmitt J., Heitz V., Sour A., Bolze F., Ftouni H., Nicoud J.F., Flamigni L., Ventura B. (2015). Diketopyrrolopyrrole-porphyrin conjugates with high two-photon absorption and singlet oxygen generation for two-photon photodynamic therapy. Angew. Chem. Int. Ed. Engl..

[126] Liu Y.-T., Yin X., Lai X.-Y., Wang X. (2020). The photophysical properties of three [M(phen)2dppz]2+ (M=Ru and Zn) derivatives for two-photon photodynamic therapy: Insights from theoretical investigations. Dye. Pigment..

[127] Hess J., Huang H., Kaiser A., Pierroz V., Blacque O., Chao H., Gasser G. (2017). Evaluation of the Medicinal Potential of Two Ruthenium(II) Polypyridine Complexes as One- and Two-Photon Photodynamic Therapy Photosensitizers. Chemistry.

[128] Mazzone G., Russo N., Sicilia E. (2013). Theoretical investigation of the absorption spectra and singlet-triplet energy gap of positively charged tetraphenylporphyrins as potential photodynamic therapy photosensitizers. Can. J. Chem..

[129] Alberto M.E., De Simone B.C., Mazzone G., Sicilia E., Russo N. (2015). The heavy atom effect on Zn(ii) phthalocyanine derivatives: A theoretical exploration of the photophysical properties. Phys. Chem. Chem. Phys..

[130] Schmitt J., Heitz V., Jenni S., Sour A., Bolze F., Ventura B. (2017). π-extended porphyrin dimers as efficient near-infrared emitters and two-photon absorbers. Supramol. Chem..

[131] Alam M.M., Bolze F., Daniel C., Flamigni L., Gourlaouen C., Heitz V., Jenni S., Schmitt J., Sour A., Ventura B. (2016). π-Extended diketopyrrolopyrrole-porphyrin arrays: One- and two-photon photophysical investigations and theoretical studies. Phys. Chem. Chem. Phys..

[132] Gouterman M., Dolphin D. (1978). Optical spectra and electronic structure of porphyrins and related rings. The Porphyrins.

[133] Yao D., Zhang X., Triadon A., Richy N., Mongin O., Blanchard-Desce M., Paul F., Paul-Roth C.O. (2017). New Conjugated meso-Tetrafluorenylporphyrin-Cored Derivatives as Fluorescent Two-Photon Photosensitizers for Singlet Oxygen Generation. Chem.-A Eur. J..

[134] Zhang X., Abid S., Shi L., Sun Z., Mongin O., Blanchard-Desce M., Paul F., Paul-Roth C.O. (2018). New conjugated meso-tetrathienylporphyrin-cored derivatives as two-photon photosensitizers for singlet oxygen generation. Dye. Pigment..

[135] Zhou Y., Cheung Y.K., Ma C., Zhao S., Gao D., Lo P.C., Fong W.P., Wong K.S., Ng D.K.P. (2018). Endoplasmic Reticulum-Localized Two-Photon-Absorbing Boron Dipyrromethenes as Advanced Photosensitizers for Photodynamic Therapy. J. Med. Chem..

[136] Awuah S.G., You Y. (2012). Boron dipyrromethene (BODIPY)-based photosensitizers for photodynamic therapy. RSC Adv..

[137] Li M., Tian R., Fan J., Du J., Long S., Peng X. (2017). A lysosome-targeted BODIPY as potential NIR photosensitizer for photodynamic therapy. Dye. Pigment..

[138] Tang Q., Si W., Huang C., Ding K., Huang W., Chen P., Zhang Q., Dong X. (2017). An aza-BODIPY photosensitizer for photoacoustic and photothermal imaging guided dual modal cancer phototherapy. J. Mater. Chem. B.

[139] Yue Y., Huo F., Cheng F., Zhu X., Mafireyi T., Strongin R.M., Yin C. (2019). Functional synthetic probes for selective targeting and multi-analyte detection and imaging. Chem. Soc. Rev..

[140] Kang J., Huo F., Chao J., Yin C. (2018). Nitroolefin-based BODIPY as a novel water-soluble ratiometric fluorescent probe for detection of endogenous thiols. Spectrochim. Acta A Mol. Biomol. Spectrosc..

[141] Kang J., Huo F., Ning P., Meng X., Chao J., Yin C. (2017). Two red-emission single and double ‘arms’ fluorescent materials stemed from ‘one-pot’ reaction for hydrogen sulfide vivo imaging. Sens. Actuators B Chem..

[142] Zhao Q., Yin C., Kang J., Wen Y., Huo F. (2018). A viscosity sensitive azide-pyridine BODIPY-based fluorescent dye for imaging of hydrogen sulfide in living cells. Dye. Pigment..

[143] Yang J., Rousselin Y., Bucher L., Desbois N., Bolze F., Xu H.J. (2018). Two-photon absorption properties of BODIPYs: Units’ number from 1 to 4 versus geometry. ChemPlusChem.

[144] Xu X., Sun D., Yang J., Zhu G., Fang Y., Gros C.P., Bolze F., Xu H.-J. (2020). Truxene-BODIPY dyads and triads: Synthesis, spectroscopic characterization, one and two-photon absorption properties and electrochemistry. Dye. Pigment..

[145] Sun J., Tian M., Lin W. (2019). A two-photon excited red-emissive probe for imaging mitochondria with high fidelity and its application in monitoring mitochondrial depolarization via FRET. Analyst.

[146] Wang H., Fang B., Kong L., Li X., Feng Z., Wu Y., Uvdal K., Hu Z. (2018). A novel Schiff base derivative: Synthesis, two-photon absorption properties and application for bioimaging. Spectrochim. Acta A Mol. Biomol. Spectrosc..

[147] Zheng Y., Sun S., Xu L., Ni S., Liu W., Huang B., Huang Q., Zhang Q., Lu F., Li M.-D. (2019). Arylamine-coumarin based donor-acceptor dyads: Unveiling the relationship between two-photon absorption cross-section and lifetime of singlet excited state intramolecular charge separation. Dye. Pigment..

[148] Cai Z.-B., Liu S.-S., Li B., Dong Q.-J., Liu Z.-L., Zheng M., Li S.-L., Tian Y.-P., Chen L.-J., Ye Q. (2019). Linear and V-shaped carbazole-based molecules functionalized by cyano acceptors and diversified donors: Synthesis, single- and two-photon related photophysical properties. Dye. Pigment..

[149] Wang Y., Jiang Y., Liu D., Wang Y., Wang G., Hua J. (2018). Ultrafast responses of two V-shaped compounds with a reverse conjugated structural configuration: An investigation of the reason for the enhanced two-photon absorption cross-section. Appl. Phys. B.

[150] Xu S., Zhu Y., Li R., Su J., Li S., Zhou H., Wu J., Tian Y. (2016). Thiophene-based pyridine derivatives: Synthesis, crystal structures, two-photon absorption properties and bio-imaging applications in the near-IR region. New J. Chem..

[151] Tao J., Sun D., Sun L., Li Z., Fu B., Liu J., Zhang L., Wang S., Fang Y., Xu H. (2019). Tuning the photo-physical properties of BODIPY dyes: Effects of 1, 3, 5, 7- substitution on their optical and electrochemical behaviours. Dye. Pigment..

[152] Zhou Y., Chen Y.-Z., Cao J.-H., Yang Q.-Z., Wu L.-Z., Tung C.-H., Wu D.-Y. (2015). Dicyanoboron diketonate dyes: Synthesis, photophysical properties and bioimaging. Dye. Pigment..

[153] Xue P., Wang P., Chen P., Yao B., Gong P., Sun J., Zhang Z., Lu R. (2017). Bright persistent luminescence from pure organic molecules through a moderate intermolecular heavy atom effect. Chem. Sci..

[154] Xiong T., Li M., Zhao X., Zou Y., Du J., Fan J., Peng X. (2020). Functional two-photon cationic targeted photosensitizers for deep-seated tumor imaging and therapy. Sens. Actuators B Chem..

[155] Morgan J., Oseroff A.R. (2001). Mitochondria-based photodynamic anti-cancer therapy. Adv. Drug Del. Rev..

[156] Kessel D., Luo Y. (1998). Mitochondrial photodamage and PDT-induced apoptosis. J. Photochem. Photobiol. B Biol..

[157] Qi J., Sun C., Li D., Zhang H., Yu W., Zebibula A., Lam J.W., Xi W., Zhu L., Cai F. (2018). Aggregation-induced emission luminogen with near-infrared-II excitation and near-infrared-I emission for ultradeep intravital two-photon microscopy. Acs Nano.

[158] Zhu M., Zhang J., Zhou Y., Xing P., Gong L., Su C., Qi D., Du H., Bian Y., Jiang J. (2018). Two-Photon Excited FRET Dyads for Lysosome-Targeted Imaging and Photodynamic Therapy. Inorg. Chem..

[159] Duan X., Jiang X.F., Hu D., Liu P., Li S., Huang F., Ma Y., Xu Q.H., Cao Y. (2018). Red emitting conjugated polymer based nanophotosensitizers for selectively targeted two-photon excitation imaging guided photodynamic therapy. Nanoscale.

[160] Redmond R.W., Gamlin J.N. (1999). A Compilation of Singlet Oxygen Yields from Biologically Relevant Molecules. Photochem. Photobiol..

[161] Jiang J., Hu D., Hanif M., Li X., Su S., Xie Z., Liu L., Zhang S., Yang B., Ma Y. (2016). Twist Angle and Rotation Freedom Effects on Luminescent Donor-Acceptor Materials: Crystal Structures, Photophysical Properties, and OLED Application. Adv. Opt. Mater..

[162] Han C., Jiang S., Qiu J., Guo H., Yang F. (2019). A diphenylacrylonitrile conjugated porphyrin with near-infrared emission by AIE–FRET. New J. Chem..

[163] Zhang L.P., Li X., Liu T., Kang L., Huang X., Zhao Y. (2020). A water-soluble pyrazino[2,3-g]quinoxaline photosensitizer for high-efficiency one- and two-photon excited bioimaging and photodynamic therapy. Chem. Commun..

[164] Zhang L.-P., Jiang K.-J., Li G., Zhang Q.-Q., Yang L.-M. (2014). Pyrazino[2,3-g]quinoxaline dyes for solar cell applications. J. Mater. Chem. A.

[165] Peng Q., Liu X., Qin Y., Xu J., Li M., Dai L. (2011). Pyrazino[2,3-g]quinoxaline-based conjugated copolymers with indolocarbazole coplanar moieties designed for efficient photovoltaic applications. J. Mater. Chem..

[166] Mastalerz M., Fischer V., Ma C.Q., Janssen R.A., Bauerle P. (2009). Conjugated oligothienyl dendrimers based on a pyrazino[2,3-g]quinoxaline core. Org. Lett..

[167] Hu B.L., Zhang K., An C., Schollmeyer D., Pisula W., Baumgarten M. (2018). Layered Thiadiazoloquinoxaline-Containing Long Pyrene-Fused N-Heteroacenes. Angew. Chem. Int. Ed. Engl..

[168] Wu W., Guo H., Wu W., Ji S., Zhao J. (2011). Organic triplet sensitizer library derived from a single chromophore (BODIPY) with long-lived triplet excited state for triplet-triplet annihilation based upconversion. J. Org. Chem..

[169] Swaminathan S., Fowley C., Thapaliya E.R., McCaughan B., Tang S., Fraix A., Captain B., Sortino S., Callan J.F., Raymo F.M. (2015). Supramolecular nanoreactors for intracellular singlet-oxygen sensitization. Nanoscale.

[170] Li S., Jiang X.-F., Xu Q.-H. (2017). Polyfluorene based conjugated polymer nanoparticles for two-photon live cell imaging. Sci. China Chem..

[171] Duan X., Liu L., Feng F., Wang S. (2010). Cationic conjugated polymers for optical detection of DNA methylation, lesions, and single nucleotide polymorphisms. Acc Chem. Res..

[172] Feng L., Zhu C., Yuan H., Liu L., Lv F., Wang S. (2013). Conjugated polymer nanoparticles: Preparation, properties, functionalization and biological applications. Chem. Soc. Rev..

[173] Lucky S.S., Soo K.C., Zhang Y. (2015). Nanoparticles in photodynamic therapy. Chem. Rev..

[174] Shen X., Li L., Wu H., Yao S.Q., Xu Q.-H. (2011). Photosensitizer-doped conjugated polymer nanoparticles for simultaneous two-photon imaging and two-photon photodynamic therapy in living cells. Nanoscale.

[175] Tuncel D., Demir H.V. (2010). Conjugated polymer nanoparticles. Nanoscale.

[176] Hashim Z., Howes P., Green M. (2011). Luminescent quantum-dot-sized conjugated polymernanoparticles—nanoparticle formation in a miniemulsion system. J. Mater. Chem..

[177] Kandel P.K., Fernando L.P., Ackroyd P.C., Christensen K.A. (2011). Incorporating functionalized polyethylene glycol lipids into reprecipitated conjugated polymer nanoparticles for bioconjugation and targeted labeling of cells. Nanoscale.

[178] Tian Z., Yu J., Wu C., Szymanski C., McNeill J. (2010). Amplified energy transfer in conjugated polymer nanoparticle tags and sensors. Nanoscale.

[179] Wu C., McNeill J. (2008). Swelling-controlled polymer phase and fluorescence properties of polyfluorene nanoparticles. Langmuir.

[180] Jin Y., Ye F., Zeigler M., Wu C., Chiu D.T. (2011). Near-infrared fluorescent dye-doped semiconducting polymer dots. ACS Nano.

[181] Wu C., Zheng Y., Szymanski C., McNeill J. (2008). Energy Transfer in a Nanoscale Multichromophoric System: Fluorescent Dye-Doped Conjugated Polymer Nanoparticles. J. Phys. Chem. C Nanomater. Interfaces.

[182] Kuimova M.K., Yahioglu G., Ogilby P.R. (2009). Singlet oxygen in a cell: Spatially dependent lifetimes and quenching rate constants. J. Am. Chem. Soc..

[183] Li S., Shen X., Xu Q.H., Cao Y. (2019). Gold nanorod enhanced conjugated polymer/photosensitizer composite nanoparticles for simultaneous two-photon excitation fluorescence imaging and photodynamic therapy. Nanoscale.

[184] Guan Z., Polavarapu L., Xu Q.H. (2010). Enhanced two-photon emission in coupled metal nanoparticles induced by conjugated polymers. Langmuir.

[185] Jiang X.F., Pan Y., Jiang C., Zhao T., Yuan P., Venkatesan T., Xu Q.H. (2013). Excitation Nature of Two-Photon Photoluminescence of Gold Nanorods and Coupled Gold Nanoparticles Studied by Two-Pulse Emission Modulation Spectroscopy. J. Phys. Chem. Lett..

[186] Guan Z., Gao N., Jiang X.F., Yuan P., Han F., Xu Q.H. (2013). Huge enhancement in two-photon photoluminescence of Au nanoparticle clusters revealed by single-particle spectroscopy. J. Am. Chem. Soc..

[187] Shen X., Li S., Li L., Yao S.Q., Xu Q.H. (2015). Highly efficient, conjugated-polymer-based nano-photosensitizers for selectively targeted two-photon photodynamic therapy and imaging of cancer cells. Chemistry.

[188] Li S., Shen X., Li L., Yuan P., Guan Z., Yao S.Q., Xu Q.H. (2014). Conjugated-polymer-based red-emitting nanoparticles for two-photon excitation cell imaging with high contrast. Langmuir.

[189] Arnbjerg J., Jimenez-Banzo A., Paterson M.J., Nonell S., Borrell J.I., Christiansen O., Ogilby P.R. (2007). Two-photon absorption in tetraphenylporphycenes: Are porphycenes better candidates than porphyrins for providing optimal optical properties for two-photon photodynamic therapy?. J. Am. Chem. Soc..

[190] Karotki A., Drobizhev M., Kruk M., Spangler C., Nickel E., Mamardashvili N., Rebane A. (2003). Enhancement of two-photon absorption in tetrapyrrolic compounds. J. Opt. Soc. Am. B.

[191] Pu K.-Y., Liu B. (2011). Bioimaging: Fluorescent Conjugated Polyelectrolytes for Bioimaging (Adv. Funct. Mater. 18/2011). Adv. Funct. Mater..

[192] Zhu C., Liu L., Yang Q., Lv F., Wang S. (2012). Water-soluble conjugated polymers for imaging, diagnosis, and therapy. Chem. Rev..

[193] Wu C., Chiu D.T. (2013). Highly fluorescent semiconducting polymer dots for biology and medicine. Angew. Chem. Int. Ed. Engl..

[194] Huang X., Neretina S., El-Sayed M.A. (2009). Gold nanorods: From synthesis and properties to biological and biomedical applications. Adv. Mater..

[195] Zhang Y., Aslan K., Malyn S.N., Geddes C.D. (2006). Metal-enhanced phosphorescence (MEP). Chem. Phys. Lett..

[196] Zhang Y., Aslan K., Previte M.J., Malyn S.N., Geddes C.D. (2006). Metal-enhanced phosphorescence: Interpretation in terms of triplet-coupled radiating plasmons. J. Phys. Chem. B.

[197] Zhang Y., Aslan K., Previte M.J., Geddes C.D. (2008). Plasmonic engineering of singlet oxygen generation. Proc. Natl. Acad. Sci. USA.

[198] Cheng D., Xu Q.H. (2007). Separation distance dependent fluorescence enhancement of fluorescein isothiocyanate by silver nanoparticles. Chem. Commun..

[199] Labouret T., Audibert J.F., Pansu R.B., Palpant B. (2015). Plasmon-Assisted Production of Reactive Oxygen Species by Single Gold Nanorods. Small.

[200] Keyvan Rad J., Mahdavian A.R., Khoei S., Shirvalilou S. (2018). Enhanced Photogeneration of Reactive Oxygen Species and Targeted Photothermal Therapy of C6 Glioma Brain Cancer Cells by Folate-Conjugated Gold-Photoactive Polymer Nanoparticles. ACS Appl. Mater. Interfaces.

[201] Zhao T., Li L., Li S., Jiang X.-F., Jiang C., Zhou N., Gao N., Xu Q.-H. (2019). Gold nanorod-enhanced two-photon excitation fluorescence of conjugated oligomers for two-photon imaging guided photodynamic therapy. J. Mater. Chem. C.

[202] Dykman L.A., Khlebtsov N.G. (2011). Gold Nanoparticles in Biology and Medicine: Recent Advances and Prospects. Acta Nat..

[203] Giljohann D.A., Seferos D.S., Daniel W.L., Massich M.D., Patel P.C., Mirkin C.A. (2010). Gold nanoparticles for biology and medicine. Angew. Chem. Int. Ed. Engl..

[204] Stranik O., McEvoy H., McDonagh C., MacCraith B. (2005). Plasmonic enhancement of fluorescence for sensor applications. Sens. Actuators B Chem..

[205] Khatua S., Paulo P.M., Yuan H., Gupta A., Zijlstra P., Orrit M. (2014). Resonant plasmonic enhancement of single-molecule fluorescence by individual gold nanorods. ACS Nano.

[206] Nooney R.I., Stranik O., McDonagh C., MacCraith B.D. (2008). Optimization of plasmonic enhancement of fluorescence on plastic substrates. Langmuir.

[207] Erwin W.R., MacKenzie R.C., Bardhan R. (2018). Understanding the limits of plasmonic enhancement in organic photovoltaics. J. Phys. Chem. C.

[208] Zeng S., Yu X., Law W.-C., Zhang Y., Hu R., Dinh X.-Q., Ho H.-P., Yong K.-T. (2013). Size dependence of Au NP-enhanced surface plasmon resonance based on differential phase measurement. Sens. Actuators B Chem..

[209] Law W.-C., Yong K.-T., Baev A., Prasad P.N. (2011). Sensitivity improved surface plasmon resonance biosensor for cancer biomarker detection based on plasmonic enhancement. ACS Nano.

[210] Chance R., Prock A., Silbey R. (1978). Molecular fluorescence and energy transfer near interfaces. Adv. Chem. Phys..

[211] Chen H., Shao L., Li Q., Wang J. (2013). Gold nanorods and their plasmonic properties. Chem. Soc. Rev..

[212] Giannini V., Fernandez-Dominguez A.I., Heck S.C., Maier S.A. (2011). Plasmonic nanoantennas: Fundamentals and their use in controlling the radiative properties of nanoemitters. Chem. Rev..

[213] Kinkhabwala A., Yu Z., Fan S., Avlasevich Y., Müllen K., Moerner W.E. (2009). Large single-molecule fluorescence enhancements produced by a bowtie nanoantenna. Nat. Photonics.

[214] Jeong Y., Kook Y.M., Lee K., Koh W.G. (2018). Metal enhanced fluorescence (MEF) for biosensors: General approaches and a review of recent developments. Biosens. Bioelectron..

[215] Zhao T., Yu K., Li L., Zhang T., Guan Z., Gao N., Yuan P., Li S., Yao S.Q., Xu Q.-H. (2014). Gold nanorod enhanced two-photon excitation fluorescence of photosensitizers for two-photon imaging and photodynamic therapy. ACS Appl. Mater. Interfaces.

[216] Lakowicz J.R. (2005). Radiative decay engineering 5: Metal-enhanced fluorescence and plasmon emission. Anal. Biochem..

[217] Fothergill S.M., Joyce C., Xie F. (2018). Metal enhanced fluorescence biosensing: From ultra-violet towards second near-infrared window. Nanoscale.

[218] Malola S., Lehtovaara L., Enkovaara J., Hakkinen H. (2013). Birth of the localized surface plasmon resonance in monolayer-protected gold nanoclusters. ACS Nano.

[219] McLean A., Wang R., Huo Y., Cooke A., Hopkins T., Potter N., Li Q., Isaac J., Haidar J., Jin R. (2020). Synthesis and Optical Properties of Two-Photon-Absorbing Au25(Captopril)18-Embedded Polyacrylamide Nanoparticles for Cancer Therapy. ACS Appl. Nano Materials.

[220] Kuruppuarachchi M., Savoie H., Lowry A., Alonso C., Boyle R.W. (2011). Polyacrylamide nanoparticles as a delivery system in photodynamic therapy. Mol. Pharm..

[221] Tang W., Xu H., Park E.J., Philbert M.A., Kopelman R. (2008). Encapsulation of methylene blue in polyacrylamide nanoparticle platforms protects its photodynamic effectiveness. Biochem. Biophys. Res. Commun..

[222] Olesiak-Banska J., Waszkielewicz M., Matczyszyn K., Samoc M. (2016). A closer look at two-photon absorption, absorption saturation and nonlinear refraction in gold nanoclusters. RSC Adv..

[223] Reuveni T., Motiei M., Romman Z., Popovtzer A., Popovtzer R. (2011). Targeted gold nanoparticles enable molecular CT imaging of cancer: An in vivo study. Int. J. Nanomed..

[224] Zhang X.D., Luo Z., Chen J., Song S., Yuan X., Shen X., Wang H., Sun Y., Gao K., Zhang L. (2015). Ultrasmall glutathione-protected gold nanoclusters as next generation radiotherapy sensitizers with high tumor uptake and high renal clearance. Sci. Rep..

[225] Wang J., Zhuo X., Xiao X., Mao R., Wang Y., Wang J., Liu J. (2019). AlPcS-loaded gold nanobipyramids with high two-photon efficiency for photodynamic therapy in vivo. Nanoscale.

[226] Meshalkin Y.P., Chunosova S.S. (2005). Two-photon absorption cross section of aluminium phthalocyanine excited by a femtosecond Ti: Sapphire laser. Quantum Electron..

[227] Nawrot K.C., Wawrzynczyk D., Bezkrovnyi O., Kepinski L., Cichy B., Samoc M., Nyk M. (2020). Functional CdS-Au Nanocomposite for Efficient Photocatalytic, Photosensitizing, and Two-Photon Applications. Nanomater.

[228] Bazylińska U., Zieliński W., Kulbacka J., Samoć M., Wilk K.A. (2016). New diamidequat-type surfactants in fabrication of long-sustained theranostic nanocapsules: Colloidal stability, drug delivery and bioimaging. Colloids Surf. B..

[229] Drozdek S., Szeremeta J., Lamch L., Nyk M., Samoc M., Wilk K.A. (2016). Two-Photon Induced Fluorescence Energy Transfer in Polymeric Nanocapsules Containing CdSe x S1–x/ZnS Core/Shell Quantum Dots and Zinc (II) Phthalocyanine. J. Phys. Chem. C.

[230] Wawrzyńczyk D., Bazylińska U., Lamch Ł., Kulbacka J., Szewczyk A., Bednarkiewicz A., Wilk K.A., Samoć M. (2019). Förster Resonance Energy Transfer-Activated Processes in Smart Nanotheranostics Fabricated in a Sustainable Manner. ChemSusChem.

[231] Acharya S., Sahoo S.K. (2011). PLGA nanoparticles containing various anticancer agents and tumour delivery by EPR effect. Adv. Drug Del. Rev..

[232] Maeda H. (2012). Macromolecular therapeutics in cancer treatment: The EPR effect and beyond. J. Control. Release.

[233] Tsai Y.-C., Vijayaraghavan P., Chiang W.-H., Chen H.-H., Liu T.-I., Shen M.-Y., Omoto A., Kamimura M., Soga K., Chiu H.-C. (2018). Targeted delivery of functionalized upconversion nanoparticles for externally triggered photothermal/photodynamic therapies of brain glioblastoma. Theranostics.

[234] Hamblin M.R. (2018). Upconversion in photodynamic therapy: Plumbing the depths. Dalton Trans..

[235] Liu Y., Meng X., Bu W. (2019). Upconversion-based photodynamic cancer therapy. Coord. Chem. Rev..

[236] Liu W., Zhang Y., You W., Su J., Yu S., Dai T., Huang Y., Chen X., Song X., Chen Z. (2020). Near-infrared-excited upconversion photodynamic therapy of extensively drug-resistant Acinetobacter baumannii based on lanthanide nanoparticles. Nanoscale.

[237] Yi Z., Luo Z., Qin X., Chen Q., Liu X. (2020). Lanthanide-activated nanoparticles: A toolbox for bioimaging, therapeutics, and neuromodulation. Acc. Chem. Res..

[238] González-Béjar M., Liras M., Francés-Soriano L., Voliani V., Herranz-Pérez V., Duran-Moreno M., Garcia-Verdugo J.M., Alarcon E.I., Scaiano J.C., Pérez-Prieto J. (2014). NIR excitation of upconversion nanohybrids containing a surface grafted Bodipy induces oxygen-mediated cancer cell death. J. Mater. Chem. B.

[239] Qiu H., Tan M., Ohulchanskyy T.Y., Lovell J.F., Chen G. (2018). Recent progress in upconversion photodynamic therapy. Nanomaterials.

[240] Buchner M., Calavia P.G., Muhr V., Kröninger A., Baeumner A.J., Hirsch T., Russell D.A., Marin M.J. (2019). Photosensitiser functionalised luminescent upconverting nanoparticles for efficient photodynamic therapy of breast cancer cells. Photochem. Photobiol. Sci..

